# P53 abnormalities and outcomes in colorectal cancer: a systematic review

**DOI:** 10.1038/sj.bjc.6602358

**Published:** 2005-01-25

**Authors:** A J Munro, S Lain, D P Lane

**Affiliations:** 1 Department of Surgery and Molecular Oncology, University of Dundee, Ninewells Hospital and Medical School, Dundee DD1 9SY, UK

**Keywords:** p53 gene, meta-analysis, systematic review, prognosis, prediction, colorectal neoplasms

## Abstract

We performed a systematic review of studies that investigated the effect of abnormalities of the tumour suppressor gene p53 upon prognosis in patients with colorectal cancer. The methods used to assess p53 status were immunohistochemistry (IHC), indicating abnormal accumulation of p53, and sequence analysis, indicating presence of p53 mutations (mut). We identified 168 reports, with 241 comparisons of relevant end points and survival data on 18 766 patients. We found evidence of both publication bias and heterogeneity of results. Our analysis was hampered by variability in both the assessment of p53 status and the reporting of results. We used a trim and fill method to correct for publication bias and minimised heterogeneity by using well-defined clinical subgroups for the assessment of outcomes. Overall, patients with abnormal p53 were at increased risk of death: relative risk (RR) with IHC 1.32 (95% confidence interval (c.i.) 1.23–1.42) and with mutation analysis 1.31 (95% c.i. 1.19–1.45). The adverse impact of abnormal p53 was greater in patients with lower baseline risk of dying: good prognosis RR (mut) 1.63 (95% c.i. 1.40–1.90) and poor prognosis RR (mut) 1.04 (95% c.i. 0.91–1.19). We found no effect of abnormal p53 on outcome in patients treated with chemotherapy. Abnormal p53 was associated with failure of response to radiotherapy in patients with rectal cancer: RR (mut) 1.49 (95% c.i. 1.25–1.77).

P53 is abnormal in more than 50% of human tumours (Vogelstein, 1990; Hollstein *et al*, 1991). Despite over 20 years of investigations, we do not know whether or not this finding is of any clinical significance. The primary function of the p53 protein is as a tumour suppressor. It can induce temporary cell cycle arrest, permitting time for repair of any DNA damage; it can induce apoptosis; and it can impose a permanent block on any future attempts at cell division (Lane *et al*, 1995; Hall and Lane, 1997; Balint and Vousden, 2001). In short, it can instruct cells to shape up, ship out, or forever cease dividing. Loss of these crucial functions could, in patients whose tumours contain abnormal p53, result in poorer outcomes than in patients whose tumours have fully functional p53. We can draw a clear distinction between p53 as an adverse prognostic factor (patients with abnormal p53 are more likely to die than patients with normal p53) and p53 as a predictive factor (patients with abnormal p53 are less likely to respond to a given treatment, say 5-fluorouracil-based chemotherapy, than patients whose p53 is normal). Prognostic factors tell us something of a tumour's intrinsic biological potential, and predictive factors tell us whether or not a particular treatment is likely to work or not.

The literature on p53 is vast. There are over 26 000 citations on Embase and more than 32 000 on Medline (July 2004). Despite this wealth of knowledge, there is no clear evidence that testing for abnormalities of p53 provides information that is useful in either a prognostic or a predictive sense. Studies on the prognostic value of p53 abnormalities are, for the reasons summarised in Table 1, extremely heterogeneous. Given this variety, it is not surprising that no clear answers have emerged. In this review, we have attempted to cut through some of these difficulties: we have used a systematic approach to the identification and analysis of studies and have confined our attentions to one tumour type, colorectal cancer. We have tried to define homogeneous groups of patients who address clinically important issues. We have attempted to answer a simple question: are patients whose tumours contain abnormal p53 more likely to die than patients whose p53 is normal?

## MATERIAL AND METHODS

We performed a systematic search of the literature using both Embase (from 1988) and Medline (from 1966) up to July 2004. We used the following search strategy (simplified):

1. (p53 and prog$).ab,sh,hw,ti,kw.
2. (p53 and pred$).ab,sh,hw,ti,kw.
3. combine 1 and 2
4. limit 3 to human
5. cancer.mp. [mp=ti, ab, rw, sh] or tumor.mp. [mp=ti, ab, rw, sh] or tumour.mp. [mp=ti, ab, rw, sh] or neoplasm.mp. [mp=ti, ab, rw, sh]
6. (colon or colorectal or rectum or rectal).mp. [mp=ti, ab, rw, sh]
7. combine 5 and 6
8. limit 7 to human
9. combine 8 and 4

This strategy yielded a total of 757 Embase citations and 954 Medline citations. These two sets were amalgamated, with duplicates discarded, and this produced a final set of 1169 potentially relevant citations. The titles and abstracts of these citations were carefully scrutinised and a study was considered eligible for the systematic review if it satisfied the following criteria:

Full publication with details of methods available.
Used human material.
Contained data on assessment of p53 status.
Contained outcome data for patients with colorectal cancer according to p53 status (outcomes were overall survival; tumour response; development of metastatic disease).
In studies reporting survival, there was a minimum of 6 months follow-up.
Sufficient detail provided to permit extraction of data for numerical analysis.

A uniform protocol was used to extract data from the full publication. Events were negatively framed (death, no response, metastatic disease). The clinical context was defined as follows: local preoperative treatment for rectal cancer, postsurgical adjuvant and advanced disease (local and/or metastatic). The primary tumour site was coded as: colon, rectum and colorectal, not otherwise specified. The methods used to assess p53 status were classified as either mutation analysis or immunohistochemistry (IHC). The analyses of mutations used single-strand conformational polymorphism or polymerase chain reaction with sequencing. The number of exons analysed was recorded for each such study. For the immunohistochemical studies, we recorded antibody used, type of specimen (fresh fixed, fresh unfixed, fixed archival) and the criterion used to define abnormal accumulation of p53.

Survival data were extracted using scanned images of published curves. The number of survivors at the maximal reliable time point was estimated using measurements from a planimetric software program, Mouseyes (Taylor, 2001). The maximum reliable time point was determined by inspection of the survival curves and was never more than twice the median survival time: values ranged from 2 to 14 years with a median of 5 years.

The data were analysed using Stata software version 7 (Stata Corporation, College Station, TX, USA). Unless otherwise specified, all analyses were performed using the random effects method (DerSimonian and Laird, 1986). We estimated absolute rate differences and relative risks (RR) for each study. We have used 95% confidence intervals (c.i.) throughout. Heterogeneity was assessed using the standard approach based on a *χ*^2^ distribution for the parameter *Q*, the measure of heterogeneity in a random effects model (DerSimonian and Laird, 1986). We anticipated that heterogeneity would cause problems with the legitimate pooling of results. We were also concerned that treatment might act as hidden confounder and so, in advance, specified that we would investigate the part played by p53 in influencing the following clinical outcomes:

Response to radiotherapy (with or without chemotherapy) in patients with rectal cancer.
Survival, and response to treatment, in patients with advanced disease.
Survival in patients not treated with chemotherapy after curative surgical treatment.
Survival in patients treated with adjuvant chemotherapy after curative surgical treatment.
Survival in patients for whom there was no information as to whether or not they had been treated with chemotherapy after curative surgical treatment.
Development of metastatic disease in patients with apparently localised disease.

Publication bias was assessed using the methods proposed by Begg (Begg and Mazumdar, 1994) and by Egger (Egger *et al*, 1997). The trim and fill technique (Duval and Tweedie, 2000) was used to investigate the impact of any bias that was suggested. We performed limited regression analyses using the method suggested by Thompson and Sharp (1999).

We assessed any bias in data extraction by carrying out regression analysis of our estimate of RR against the RR or hazard ratio (HR) reported by the original investigators, where available. Any systematic bias would produce an intercept value greater than zero.

## RESULTS

The search strategy identified 227 relevant papers and abstracts. Since some papers reported data on more than one end point, we were able to identify 287 comparisons. The reports included data from a total of 33 648 patients. Once we had eliminated duplicate publication of the same data, there were 168 papers, with 241 comparisons, left. These studies published survival data on 18 766 patients, response data on 1514 patients and, for 1066 patients, data on the effect of abnormal p53 upon the development of metastatic disease were available.

In 61 studies, we were able to compare the extracted RR with the RR or HR reported by the authors. There was good agreement (Spearman's correlation coefficient 0.70, *P*<0.0001). The intercept of the regression line on the *Y*-axis (RR) was 0.01.

**Figure 1:**
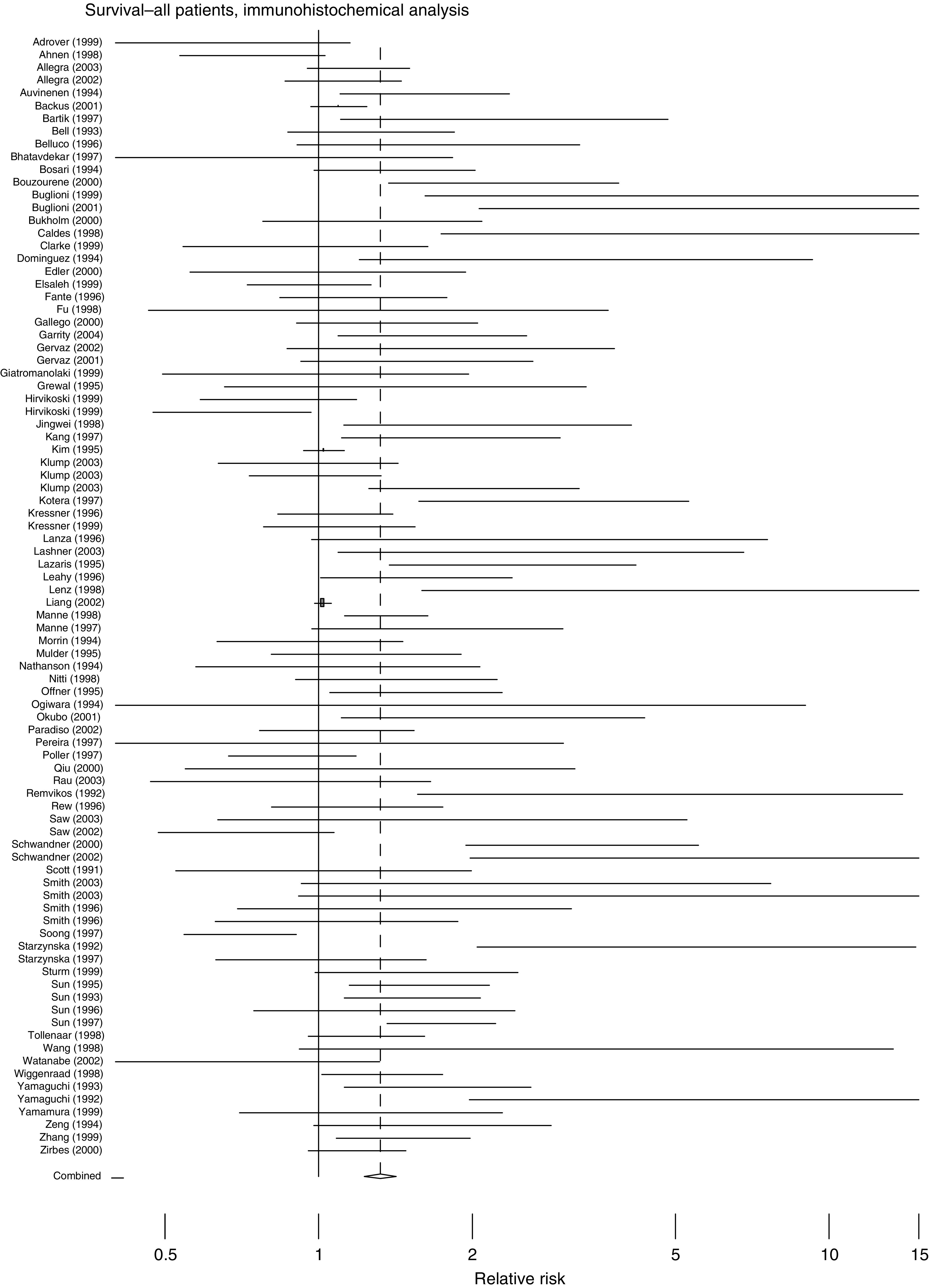
Forest plot for all studies, with survival as outcome, using IHC to define p53 status. Log RR – log relative risk, values >1.0 indicate that abnormal p53 is associated with increased hazard, that is, lower survival. Each study is shown with its 95% c.i. The size of the square symbol is proportional to the weight assigned to the study in the pooled estimate using a random effects model.

**Figure 2:**
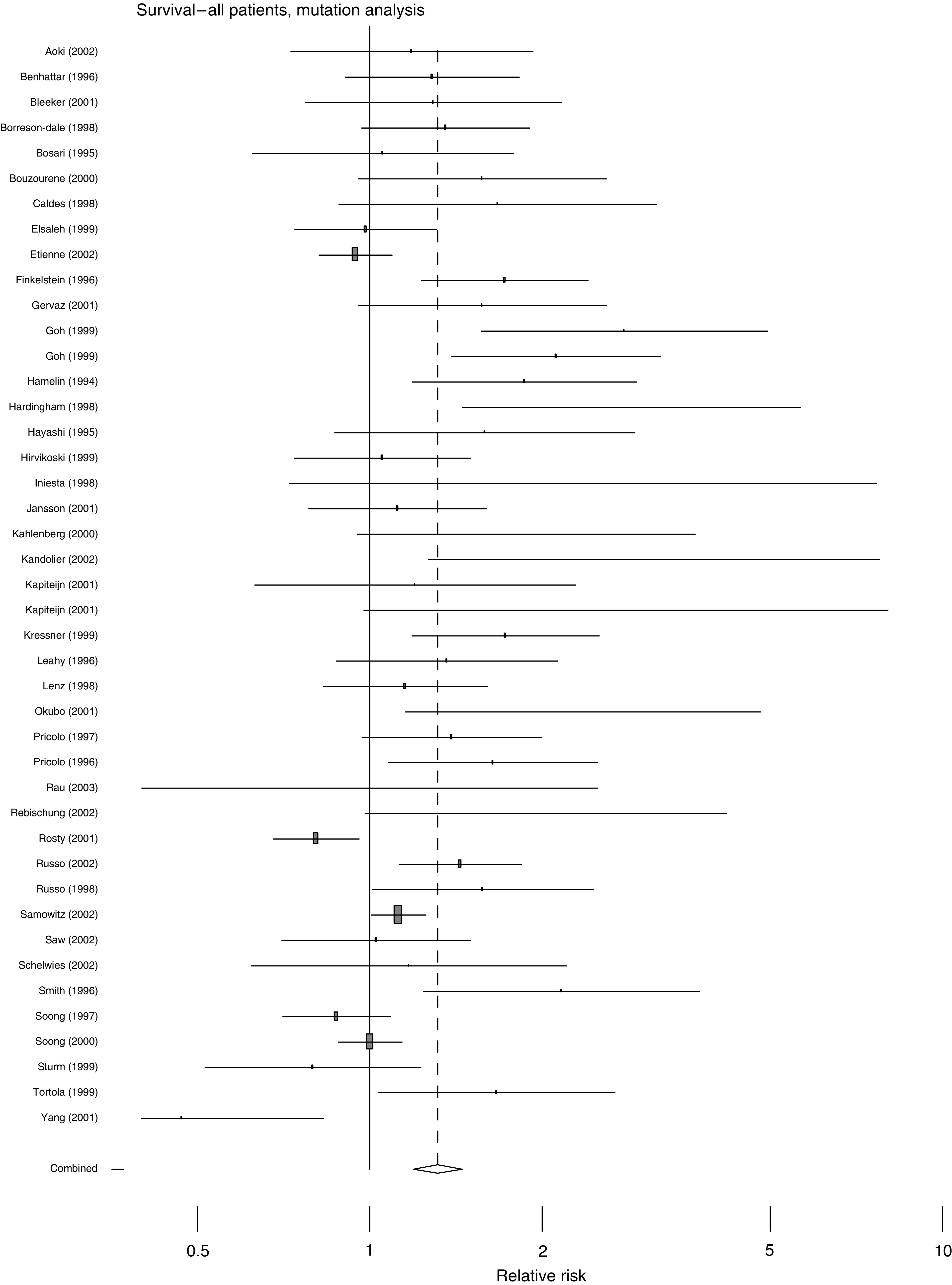
Forest plot for all studies, with survival as outcome, using analysis of sequence data to define p53 status.

**Figure 3:**
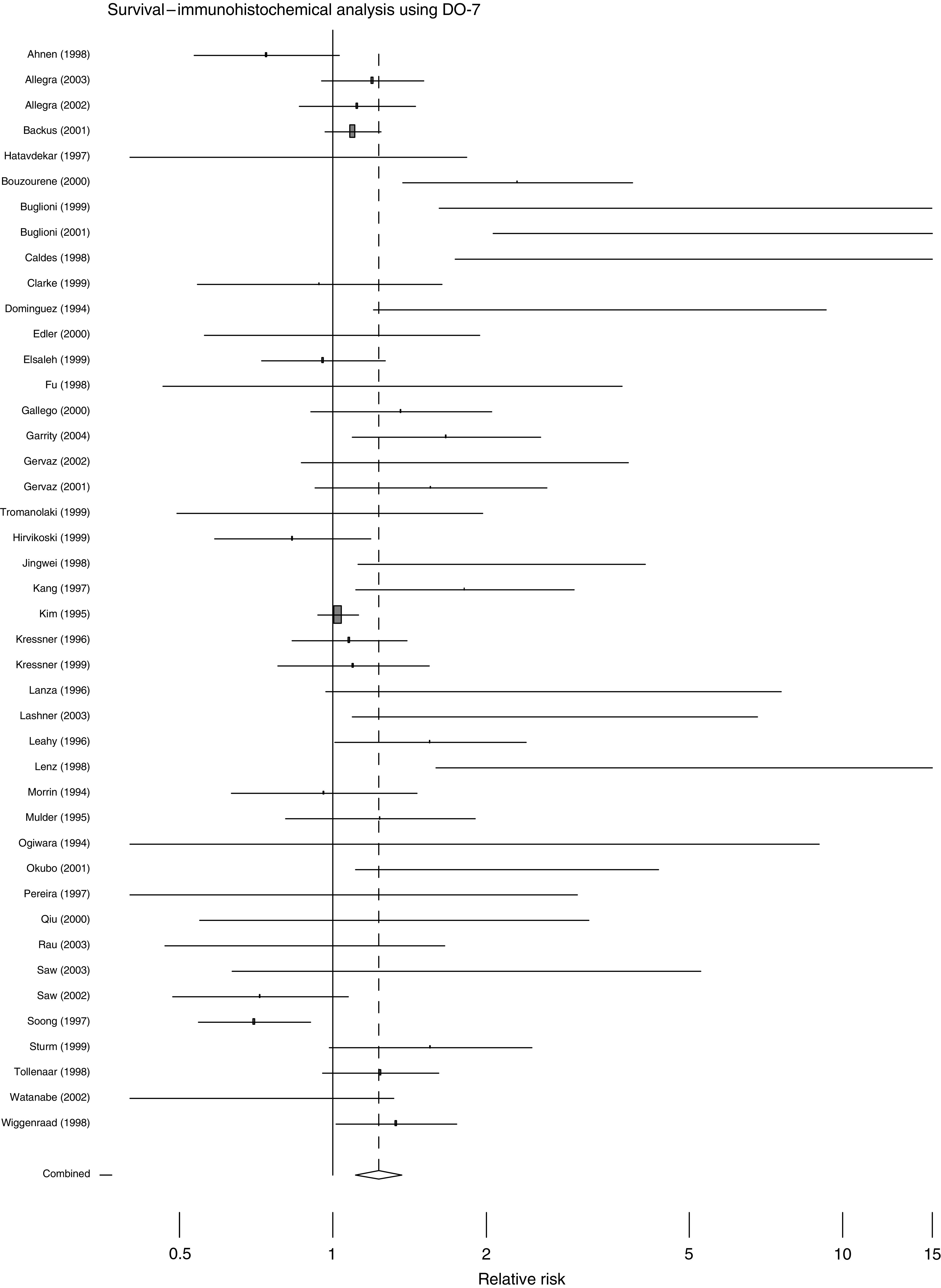
Forest plot for all studies, with survival as outcome, using IHC with the DO-7 antibody to define p53 status.

**Figure 4:**
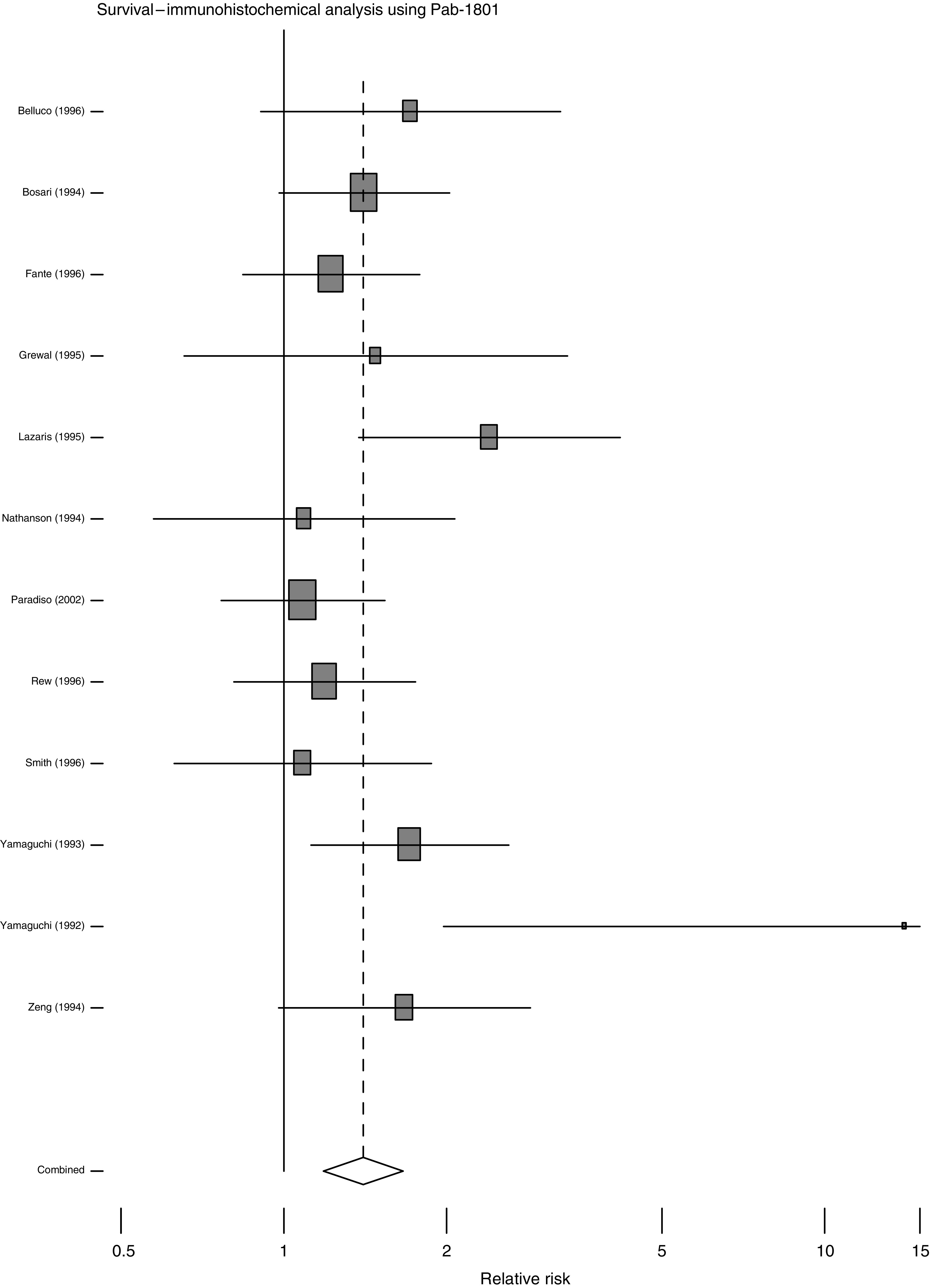
Forest plot for all studies, with survival as outcome, using IHC with the Pab-1801 antibody to define p53 status.

The overall effects of abnormal p53 upon survival are shown in Figures 1, 2, 3 and 4. The majority of the immunohistochemical studies used either DO-7 or Pab-1801 as the antibody for identifying abnormal accumulation of p53 and so results for these antibodies are shown separately. Table 2 shows the accompanying numerical data. As anticipated, these analyses, with the exception of IHC using Pab-1801, show considerable heterogeneity and so we performed a series of more restricted analyses. We found no evidence for any relationship between the criterion used to define ‘positive’ by IHC and outcome. For example, using the DO-7 antibody, and with survival as outcome, the absolute rate difference was 13.4% (95% c.i. 2.4–24.5%) when the criterion was set at >1% cells positive, and 13.5% (95% c.i. 6.9% to 20.1%) when a cutoff value of >10% positivity was used.

Table 3 shows analyses by tumour site and, in addition, shows data on publication bias and its likely effect upon the estimates of effect. There is clear evidence of publication bias and, as expected, its effect is to exaggerate any estimate of the adverse effect of p53 upon survival. The estimates of RR are inflated by 0.20, which corresponds, in this population of patients, to overestimating the absolute rate difference by about 10%.

**Figure 5:**
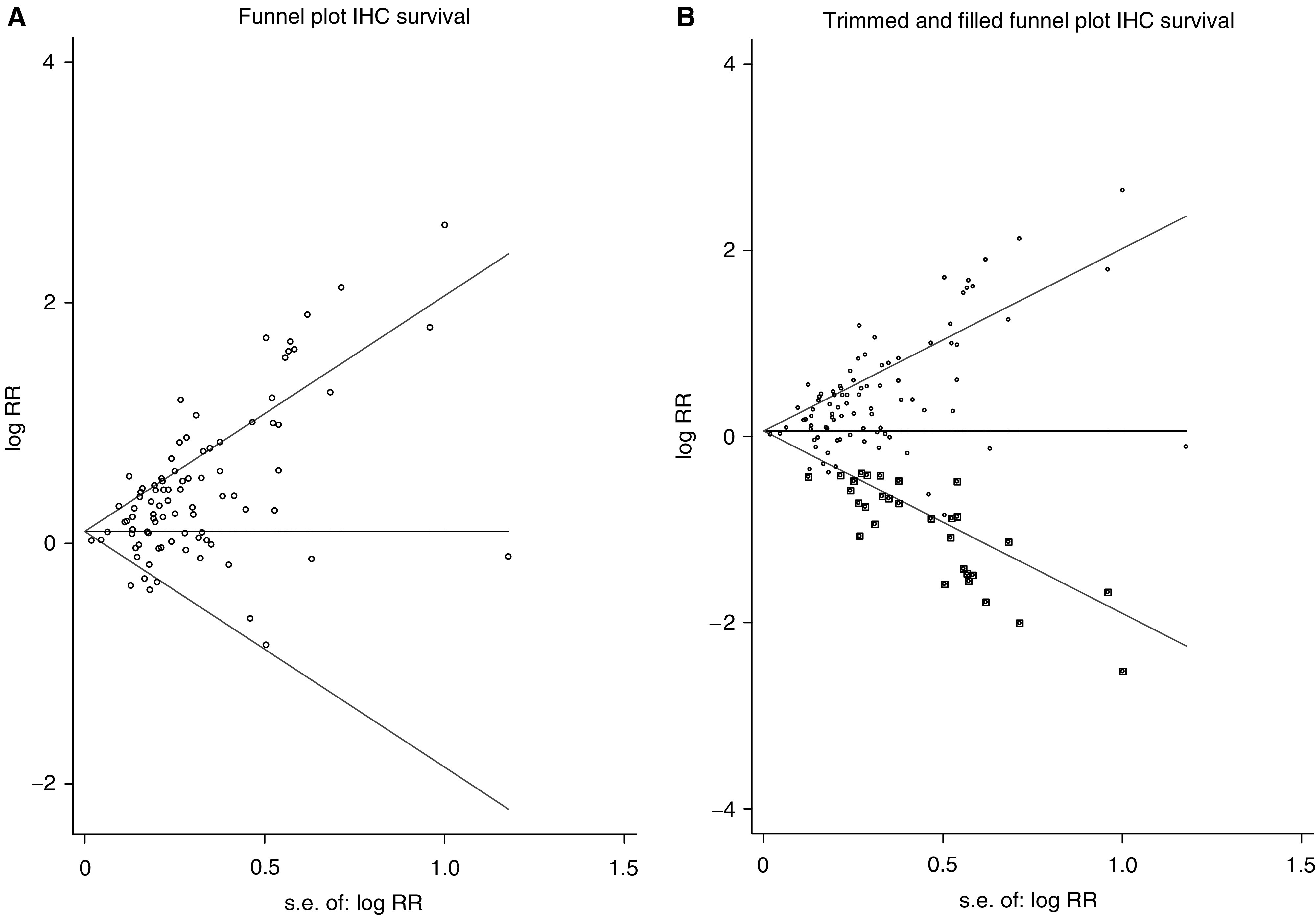
log RR – log relative risk; s.e. – standard error (**A**) Funnel plot of data on survival in studies using IHC. As discussed in the text, there is evidence of publication bias – asymmetry due to lack of negative studies with high standard error and low statistical power. (**B**) Funnel plot of the same group of studies after trimming and filling. The dummy studies are indicated by circles within squares, and the genuine studies, as in (**A**), by circles.

Figure 5A and B are funnel plots illustrating the trim and fill approach to publication bias (Duval and Tweedie, 2000). The round circles show individual studies on a plot of estimates of the log risk ratio *vs* its standard error. The circles with squares around them, 30 in number, in Figure 5B indicate dummy studies invented by the trim and fill method to counteract bias. Points lying above zero on the *Y*-axis are positive studies, and points lying below are negative studies. The further to the right a point lies, the lower the statistical power of the study it represents. If publication bias did not apply, all points would lie symmetrically about the central measure of effect, the horizontal line which, in this case, is just above zero. The plot clearly shows that small negative studies are under-represented (fewer circles towards the bottom right of the graph in Figure 5A) and the use of the dummy studies to compensate for this deficiency.

The data in Table 4 are from analyses restricted to clinically useful categories. This approach considerably reduces heterogeneity, or at least the statistical estimate thereof, and also reduces the impact of any publication bias.

Using regression analysis, we investigated the possibility that treatment with chemotherapy might confound estimates of effect in studies of patients treated by curative surgical resection. We used the percentage of patients in each study known to have been treated with chemotherapy as the predictive variable. We could find no significant effect: in studies using IHC, the regression coefficient was 0.0023 (−0.0031 to +0.0077; *P*=0.41) and in studies using analysis of mutations, the coefficient was 0.0012 (−0.0031 to +0.0056; *P*=0.577). These findings are consistent with the results in Table 4, which show no discernible effect of chemotherapy upon the estimate of RR.

**Figure 6:**
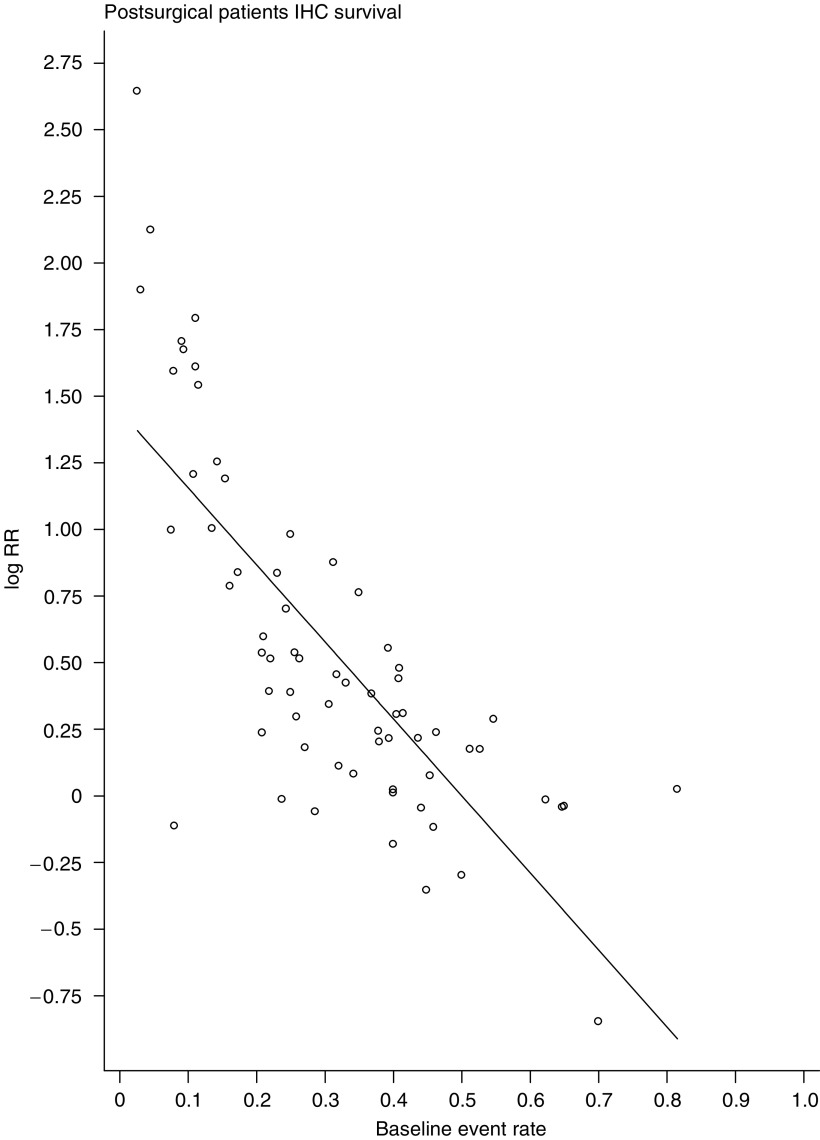
Plot of the estimate of log RR (log relative risk) against baseline event rate in studies of patients operated upon for cure and whom p53 status had been assessed using IHC. Baseline event rate, for each study, is defined as the proportion of deaths in patients with normal p53.

We also used metaregression to assess whether baseline risk, defined as the risk of death in patients with normal p53, had any influence upon the effect of abnormal p53 on survival. Baseline risk had a marked influence on the adverse effect of abnormal p53 (*P*<0.0001). This applied whether p53 status had been assessed by IHC or by mutational analysis. For every 10% rise in baseline risk of death, the absolute rate difference associated with abnormal p53 decreased by 6% (95% c.i. 4–8%; *P*<0.0001) Figure 6. This effect persisted after adjustment for the percentage of patients in each study receiving chemotherapy.

The median baseline risk was 0.35. We used this value to divide groups of patients into those with good prognosis (risk of death <35%) and those with poor prognosis (risk of death >35%). Table 5 shows the effect of abnormal p53 upon outcome for patients treated by curative surgery according to baseline risk. The adverse effect of p53 upon outcome is greater in those patients whose underlying prognosis is better.

## DISCUSSION

This review epitomises the difficulties and pitfalls encountered in systematic reviews of observational studies. We found evidence for significant publication bias and, in the pooled analyses of survival, heterogeneity erodes the validity of the estimates of overall effect. Figures 1, 2, 3 and 4 are best interpreted as a convenient means of showing the pattern of results. They do not provide any precise estimate of overall effect.

The presence of publication bias (Tables 3 and 4) means that, according to the results of the compensatory trim and fill method (Duval and Tweedie, 2000), unadjusted estimates of the adverse effect of abnormal p53 should be scaled downwards: by about 0.22 for RR and by around 10% for absolute rate difference. However, this method may overestimate the magnitude of any publication bias (Sterne and Egger, 2000) and so the figures, 0.22 and 10%, should be regarded as maximal estimates of the effect of publication bias. There is no clear evidence that the adverse effect of abnormal p53 upon outcome depends upon the location of the primary tumour (Table 3). The adjusted estimates of RR are between 1.16 and 1.19 for rectal tumours and around 1.13 for colonic tumours. This issue is distinct from the difference in the rate of p53 abnormalities, which is commoner in left-sided, as opposed to right-sided, tumours (Soong *et al*, 2000). In brief, p53 abnormalities may be commoner in rectal tumours, but the adverse consequences of any p53 mutation are of similar magnitude, regardless of whether the primary tumour is in the colon or in the rectum.

By restricting the pooled analyses to well-defined clinical questions (Table 4), we were able to decrease the apparent heterogeneity and this, together with a reduction in publication bias, means that we can draw some reasonably robust conclusions from the data. Mutant p53, as detected by sequence analysis, predicts treatment failure in patients with rectal cancer treated with radiotherapy or chemoradiation (RR 1.49; c.i. 1.25–1.77). Abnormal p53, as detected by IHC, has no predictive value in this group of patients (RR 1.15; c.i. 0.88–1.52). Abnormalities in p53, whether assessed immunohistochemically or by sequence analysis, appear to be of no value in predicting response to chemotherapy alone (Table 4). The effects of p53, and its abnormalities, on the response of tumours to cytotoxic drugs, radiation, and chemoradiation are complex (Blandino *et al*, 1999; Bunz *et al*, 1999; El-Deiry, 2003; Fei and El-Deiry, 2003; Gudkov and Komarova, 2003), and it is, perhaps, unrealistic to expect a straightforward relationship between any abnormality of p53 and the response to treatment with chemotherapy. Another complicating factor is that polymorphisms in wild-type p53, and its regulator, MDM2 (Bond *et al*, 2004), may also affect response to treatment (Sullivan *et al*, 2004): in this respect, both ‘normal’ and ‘abnormal’ p53 are heterogeneous entities.

The results from the studies on patients treated with potentially curative surgery again suggest that abnormalities of p53 may have no significant impact upon the response of colorectal cancer to chemotherapy. This is suggested both by the data in Table 4 and by regression analysis showing that the percentage of patients within a study who were treated with adjuvant chemotherapy had no influence upon RR. Unfortunately, due to the conduct and reporting of the studies included in this systematic review, it is not possible to pursue this argument further.

The evidence on the effect of the p53 status of the primary tumour upon the likelihood of metastatic disease is conflicting. The immunohistochemical data suggest no effect (RR 0.92; c.i. 0.61–1.39), whereas the more limited data using analysis of mutations suggest that abnormal p53 may significantly increase the risk of the development of metastatic disease (RR 1.67; c.i. 1.21–2.30). This is clearly an area that warrants further investigation.

The most important conclusion to emerge from this review is the recognition that, in patients treated with curative surgery, the baseline risk of death is an important factor in determining the magnitude of the adverse effect on survival associated with abnormal p53 (Table 5 and Figure 6). Abnormal p53 had more of an impact on survival in patients whose underlying prognosis was better. This suggests that abnormalities in p53 may have an independently adverse impact upon prognosis. This question is best addressed prospectively and could be incorporated into the design of clinical trials. Adjusted analyses, with p53 status as a separate variable, would indicate whether abnormalities in p53 have an adverse effect over and above that associated with known prognostic factors, such as clinicopathological stage.

Only one previous review (Petersen *et al*, 2001) has addressed the question of whether or not abnormalities of p53 affect outcome in patients with colorectal cancer. Their review included 28 studies, involving 4416 patients. Their results suggested that, overall, abnormal p53 had an adverse effect on survival, but that this effect was by no means consistent. They concluded that: ‘p53 remains an investigational parameter’. Our own review takes things a little further, but not as far as the investment of resources in investigating the prognostic value of p53 in colorectal cancer should have enabled us to go. It is salutary to realise that, worldwide, we have studied over 18 000 patients and spent, at a conservative estimate, over £6.5 million on investigating abnormal p53 in colorectal cancer; yet we have found out very little that we can put to clinical use.

One reason for this is a certain naïveté concerning techniques for assessing abnormalities in p53. Positive IHC does not necessarily imply that p53 is functionally inactive nor does the absence of a demonstrable mutation mean that p53 is fully active. The use of IHC to identify mutant p53 is based on the assumption that abnormal p53 cannot act as a transcription factor. It cannot, therefore, switch on its own, MDM2 mediated, destruction. And so it accumulates. This may be an oversimplification. Accumulation of p53, as detected immunohistochemically, may not inevitably imply the presence of p53 that, through mutation, is transcriptionally inactive. Wild-type p53 might accumulate if there is amplification or overexpression of HDMX proteins (Ramos *et al*, 2001); if p53 is denied access to the nucleus (O'Brate and Giannakakou, 2003); and if p73 isoforms impair the transcriptional activity of wild-type p53 (Concin *et al*, 2004). All mutations in p53 have been treated as if they were of equal prognostic significance: an assumption that should not be taken for granted. Mutations in p53 can have a variety of effects and these include gain of function as well as loss of function. Loss of functional p53 implies an inability to undergo apoptosis or cell cycle arrest, which may, in turn, lead to genomic instability. Dominant-negative mutations will suppress the functional activity of any wild-type p53 that is present, leading to loss of normal protective mechanisms. Some p53 mutations cause gain of function that is independent of any complex formation with wild-type p53 and is associated with selective proliferative advantage (Gurova *et al*, 2003; Scian *et al*, 2004). There is good evidence that different mutations in p53 have different effects upon the sensitivity of tumours to treatment (Blandino *et al*, 1999; Dridi *et al*, 2003; Klumb *et al*, 2003). P53 has a pivotal role in cellular husbandry and the p53 protein does not operate in isolation. The actions of p53 are influenced by other members of the p53 pathway and a straightforward ‘normal is good – all mutation is bad’ argument is unlikely to apply.

There is a clear distinction between an observation that is biologically interesting and a test that is clinically useful. The former simply implies some degree of association; the latter requires a tight relationship between the biological finding and the clinical outcome. The positive predictive value (PPV) is a useful metric for assessing a prognostic or predictive factor. It is easily computed and can be expressed straightforwardly in words: ‘what proportion of subjects with a positive test result experiences the outcome of interest?’. Our results suggest that, with current methods of assessment, p53 status is a poor guide to outcome (prognosis) or response to treatment (prediction). The PPV's derived from our analysis are typically around 0.5, no better than the toss of a coin.

The problems with the design and interpretation of studies dealing with the assessment of prognostic factors have been well rehearsed (Altman and Lyman, 1998; Altman, 2001; Deeks *et al*, 2003; Riley *et al*, 2003a, 2003b; Riley *et al*, 2004). During the progress of this review, we encountered most of them. The problems listed in Table 1 remain with us. Future studies on p53 as a prognostic factor should include data on subgroups defined by clinicopathological stage, by site of tumour, and by treatment. We also need a more standardised approach to the investigation of p53 status. There is, to judge by usage, no consensus on any optimal method for the use of IHC to detect abnormal accumulation of p53; nor is there any apparent agreement on how best to identify mutations in the 11 exons of the p53 gene. Given that mutation and accumulation, as detected by IHC, may be telling us different things (Bazan *et al*, 2002), it would be sensible if future studies combined both methods of assessment. Until procedures and approaches to the investigation of p53 status are standardised, there can be no real progress:

Dix millions d'ignorances, ne font pas un savoir (Ten million errors do not leave us any the wiser; Hippolyte Taine, 1823–1898).

In the meantime, we can conclude with some degree of confidence that:

In patients with better underlying prognosis, that is, survival rates of >65% after surgery, abnormal p53 has an adverse effect on outcome.
Abnormal p53 does not affect the outcome in patients treated with 5FU-based chemotherapy.
Rectal tumours containing proven mutations in p53 are less likely to respond to radiation, or chemoradiation, than rectal cancers without evidence of mutant p53.

To the question: are patients whose tumours contain abnormal p53 more likely to die than patients whose p53 is normal?, we can only answer: sometimes.

## Figures and Tables

**Table 1:** Sources of heterogeneity in studies assessing the value of abnormal p53 as a predictive or prognostic marker

Different tumour types, different histological grades, different clinical stages
Different tissue: primary *vs* metastases
Different treatments
Different ethnic groups
Different socioeconomic groups (smoking, alcohol)
Different experimental designs: retrospective case reports → prospective studies nested within randomised trials
Different sample types: fresh–frozen, fresh–fixed, archival
Different assay techniques: IHC; mutation analysis
Different criteria for discriminating between normal and abnormal p53 (cutoff levels)
Different effects of different abnormalities of p53
Different effects on different components of the p53 pathway (no molecule is an island unto itself)

IHC=immunohistochemistry.

**Table 2:** Summary data on all comparisons with survival as the end point

**Group**	**Rate difference (95% c.i.)**	***P* het.**	***P* sig.**	**RR (95% c.i.)**	***P* het.**	***P* sig.**	**Events**	**Total**
*IHC*								
All	0.13 (0.10–0.16)	<0.0001	<0.0001	1.32 (1.23–1.42)	<0.0001	<0.0001	5087	12 257
DO-7	0.10 (0.05–1.14)	<0.0001	<0.0001	1.24 (1.12–1.37)	<0.0001	<0.0001	2566	6436
Pab-1801	0.16 (0.09–0.23)	0.05	<0.0001	1.40 (1.18–1.66)	0.132	<0.0001	511	1281
Others	0.15 (0.10–0.21)	<0.0001	<0.0001	1.43 (1.25–1.63)	<0.0001	<0.0001	2010	4540

Mutations	0.12 (0.08–0.17)	<0.0001	<0.0001	1.31 (1.19–1.45)	<0.0001	<0.0001	3128	6645

IHC=immunohistochemical detection of accumulation of p53; DO-7=studies using the DO-7 antibody; Pab-1801=studies using the antibody Pab-1801; others=all other IHC studies; mutations=studies in which p53 mutations were identified using sequencing; RR=relative risk; c.i.=confidence interval; *P* het.=the *P* value, derived from the *Q* statistic, for heterogeneity/homogeneity. The lower the *P*-value, the less likely it is that the studies in the analysis are homogeneous: low *P*-values indicate heterogeneity. *P* sig.=the *P*-value for the observed effect; events=deaths; total=total number of patients in each analysis.

**Table 3:** Summary data on survival by method and site

**Site**	**Method**	**Studies**	**RR**	**Sig**	**Het**	**Begg**	**Eggers**	**Fill**	**T&F(R)**	**T&F(F)**	**T&F het**
Rectum	IHC	16	1.29 (1.00–1.66)	0.048	<0.0001	0.685	0.295	1	1.22 (0.94–1.59) *P*=0.135	1.16 (1.03–1.30) *P*=0.014	<0.0001
	Mut	7	1.28 (1.06–1.54)	0.011	0.004	0.368	0.063	2	1.32 (0.89–1.94) *P*=0.165	1.19 (0.99–1.43) *P*=0.058	0.001

Colon	IHC	9	1.28 (1.01–1.62)	0.042	0.001	0.076	0.097	2	1.14 (0.89–1.48) *P*=0.299	1.12 (1.00–1.27) *P*=0.051	<0.0001
	Mut	6	1.39 (1.12–1.74)	0.003	0.047	1.0	0.1	3	1.15 (0.93–1.42) *P*=0.207	1.13 (1.04–1.24) *P*=0.007	0.001

Colorectal	IHC	64	1.34 (1.24–1.46)	<0.0001	<0.0001	0.007	<0.0001	24	1.12 (1.03–1.22) *P*=0.01	1.06 (1.03–1.09) *P*<0.0001	<0.0001
	Mut	30	1.28 (1.13–1.44)	<0.0001	<0.0001	0.052	<0.0001	11	1.06 (0.94–1.2) *P*=0.329	1.02 (0.97–1.08) *P*=0.414	<0.0001

IHC=immunohistochemistry; Mut=analysis of mutations using sequencing; studies=number of included studies; RR=relative risk, with 95% c.i. in brackets; Sig=*P-*value for the observed effect; Het=*P-*value for heterogeneity; Begg=*P-*value for publication bias calculated using Begg's method; Egger=*P-*value for publication bias calculated used Egger's method; fill=number of studies added by trim and fill method; T&F(R)=relative risk (and 95% c.i.) of trimmed and filled analysis calculated using assumption of random effects; T&F(F)=relative risk (and 95% c.i.) calculated using assumption of fixed effects; T&F het=*P-*value for homogeneity/heterogeneity calculated for trimmed and filled analysis.

**Table 4:** Analysis of studies dealing with clinically relevant subgroups

**Comparison**	**Method**	***N***	**RR**	**Sig**	**Het**	**Begg**	**Eggers**	**T&F**
Rectal cancer Response – XRT/chemo-RT	IHC	13	1.15 (0.88–1.52)	0.31	0.048	0.502	0.019	No change
	Mut	6	1.49 (1.25–1.77)	<0.0001	0.752	0.707	0.04	No change
Advanced diseaseResponse – chemo	IHC	9	1.11 (0.93–1.31)	0.235	0.048	0.348	0.113	1.02 (0.85–1.22) *P*=0.862
	Mut	2	0.65 (0.32–1.32)	0.231	0.144	N/A	N/A	N/A
Advanced disease Survival	IHC	7	1.09 (0.96–1.23)	0.186	0.065	0.138	0.236	1.04 (0.91–1.18) *P*=0.572
	Mut	8	0.97 (0.81–1.16)	0.736	0.007	1.00	0.665	0.92 (0.76–1.11) *P*=0.373
Postsurgical: no adjuvant chemoSurvival	IHC	11	1.28 (1.10–1.49)	0.002	0.067	0.119	0.072	No change
	Mut	4	1.67 (1.35–2.03)	<0.0001	0.471	0.089	0.089	No change
Postsurgical: adjuvant chemoSurvival	IHC	6	1.66 (1.16–2.79)	0.005	0.001	0.26	0.054	1.26 (0.84–1.91) *P*=0.264
	Mut	3	1.60 (1.27–2.02)	<0.0001	0.649	0.296	0.239	No change
Postsurgical: chemo unknownSurvival	IHC	40	1.53 (1.36–1.73)	<0.0001	<0.0001	0.002	<0.0001	1.26 (1.11–1.43) *P*<0.0001
	Mut	15	1.60 (1.30–1.94)	<0.0001	0.002	0.048	0.001	1.20 (0.98–1.48) *P*=0.073
Development of metastases	IHC	8	1.09 (0.78–1.53)	0.51	0.043	0.06	0.02	0.92 (0.61–1.39) *P*=0.682
	Mut	2	1.67 (1.21–2.30)	0.002	0.301	N/A	N/A	N/A

IHC=immunohistochemistry; Mut=analysis of mutations using sequencing; *N*=number of studies included in analysis; RR=relative risk, with 95% c.i. in brackets; sig=*P* value for the observed effect; het=*P*-value for heterogeneity; Begg=*P*-value for publication bias calculated using Begg's method; Egger=*P*-value for publication bias calculated using Egger's method; T&F=result of trimmed and filled analysis, using assumption of random effects; N/A=not applicable.

**Table 5:** Survival data from studies on patients who were considered to have had curative surgery^a^

**Group**	**Method**	**RR**	**Rate difference**	**Het.**	**T&F RR**
Good	IHC	1.89 (1.63–2.19) *P*<0.0001	0.20 (0.16–0.25) *P*<0.0001	<0.0001	1.53 (1.30–1.80)
	Mut	1.75 (1.51–2.02) *P*<0.0001	0.21 (0.16–0.26) *P*<0.0001	0.37	1.63 (1.40–1.90)

Bad	IHC	1.13 (1.03–1.24) *P*=0.009	0.06 (0.01–0.11) *P*=0.015	<0.0001	No change
	Mut	1.21 (1.07–1.37) *P*=0.003	0.11 (0.03–0.18) *P*=0.002	0.004	1.04 (0.91–1.19)

IHC=immunohistochemistry; RR=relative risk; Mut=analysis of mutations using sequencing; RR and rate difference are shown together with 95% c.i. and the *P*-value for the observed effect; Het=the *P*-value for homogeneity/heterogeneity; T&F RR=relative risk, and 95% c.i., after adjustment for publication bias using the trim and fill method calculated using a random effects model.

a The ‘good’ group involved studies in which patients with normal p53 had survival rates of over 65%; the ‘bad’ group involved studies in which patients with normal p53 had survival rates less than 65%.
