# Supplementary material for: P53 abnormalities and outcomes in colorectal cancer: a systematic review
Source: Br J Cancer. 2005 Jan 25;92(3):434–44. doi: 10.1038/sj.bjc.6602358 (PMC2362083; doi:10.1038/sj.bjc.6602358)
Supplement: Supplementary File 1 [file 92-6602358x1.pdf]

| id | study | pa | year | size with data | size overall | site | method | endpoint | context | % with abnormal p53 | antibody | criterion | from exon |
|---|---|---|---|---|---|---|---|---|---|---|---|---|---|
| 81 | adell | p | 1999 | 163 | 163 | rectum | IHC | local control | local preop | 41% | Pab-1801 | 0.05 | ********** |
| 82 | adell | p | 1999 | 85 | 85 | rectum | IHC | local control | local preop | 45% | Pab-1801 | 0.05 | ********** |
| 83 | adell | p | 1999 | 78 | 78 | rectum | IHC | local control | local preop | 37% | Pab-1801 | 0.05 | ********** |
| 44 | adrover | p | 1999 | 30 | 30 | colorectal | IHC | survival | post surg. adj. | 33% | DO-1 | 0.99 | ********** |
| 30 | ahnen | p | 1998 | 194 | 194 | colon | IHC | survival | post surg. adj. | 64% | DO-7 | 0.05 | ********** |
| 26 | allegra | p | 2003 | 700 | 700 | colon | IHC | survival | post surg. adj. | 54% | DO-7 | 0.1 | ********** |
| 20 | allegra | p | 2002 | 463 | 463 | colorectal | IHC | survival | post surg. adj. | 60% | DO-7 | 0.1 | ********** |
| 29 | allegra | p | 2003 | 700 | 700 | colon | IHC | FFS | post surg. adj. | 54% | DO-7 | 0.1 | ********** |
| 84 | aoki | p | 2002 | 68 | 68 | colorectal | mut | survival | post surg. adj. | 59% | ********** | ********** | 5 |
| 41 | auvinenen | p | 1994 | 144 | 144 | colon | IHC | survival | post surg. adj. | 69% | CM-1 | 0.2 | ********** |
| 64 | backus | p | 2001 | 29 | 29 | colorectal | IHC | survival | advanced / mets. | 21% | DO-7 | 0.25 | ********** |
| 85 | backus | p | 2001 | 30 | 29 | colorectal | IHC | response | advanced / mets. | 21% | DO-7 | 0.25 | ********** |
| 59 | baretton | p | 1996 | 95 | 95 | colorectal | IHC | FFS | post surg. adj. | 42% | DO-1 | 0.2 | ********** |
| 53 | bartik | p | 1997 | 82 | 257 | colorectal | IHC | survival | post surg. adj. | 44% | DO-1 | 0.05 | ********** |
| 86 | bell | p | 1993 | 100 | 100 | colorectal | IHC | survival | post surg. adj. | 46% | Pab421 | 0.01 | ********** |
| 78 | belluco | p | 1996 | 48 | 50 | colorectal | IHC | survival | advanced / mets. | 72% | Pab-1801 | 0.1 | ********** |
| 87 | belluco | p | 1996 | 50 | 50 | colorectal | IHC | response | advanced / mets. | 72% | Pab-1801 | 0.1 | ********** |
| 88 | benhattar | p | 1996 | 17 | 17 | colorectal | mut | survival | advanced / mets. | 47% | ********** | ********** | 5 |
| 256 | berglund | p | 2002 | 120 | 136 | colorectal | IHC | response | advanced / mets. | 60% | DO-7 | 0.05 | ********** |
| 89 | bhatavdeka | p | 1997 | 48 | 48 | colorectal | IHC | survival | post surg. adj. | 38% | DO-7 | 0.05 | ********** |
| 90 | bleeker | p | 2001 | 55 | 55 | colon | mut | survival | post surg. adj. | 44% | ********** | ********** | 5 |
| 91 | borreson-d | p | 1998 | 222 | 222 | colorectal | mut | survival | post surg. adj. | 46% | ********** | ********** | 5 |
| 92 | bosari | p | 1995 | 126 | 126 | colorectal | mut | survival | post surg. adj. | 59% | ********** | ********** | 5 |
| 93 | bosari | p | 1994 | 206 | 206 | colorectal | IHC | survival | post surg. adj. | 46% | Pab-1801 | 0.01 | ********** |
| 94 | bouzourene | p | 2000 | 122 | 122 | colorectal | mut | survival | post surg. adj. | 32% | ********** | ********** | 5 |
| 46 | bouzourene | p | 2000 | 112 | 122 | colorectal | IHC | survival | post surg. adj. | 47% | DO-7 | 0.1 | ********** |
| 95 | brett | p | 1996 | 59 | 59 | colorectal | IHC | response | advanced / mets. | 49% | DO-1 | 0.05 | ********** |
| 77 | buglioni | p | 2001 | 94 | 94 | colorectal | IHC | survival | post surg. adj. | 53% | DO-7 | 0.01 | ********** |
| 96 | buglioni | p | 1999 | 141 | 171 | colorectal | IHC | survival | post surg. adj. | 72% | DO-7 | 0.01 | ********** |
| 76 | buglioni | p | 2001 | 94 | 94 | colorectal | IHC | FFS | post surg. adj. | 53% | DO-7 | 0.01 | ********** |
| 80 | buglioni | p | 1999 | 119 | 171 | colorectal | IHC | FFS | post surg. adj. | 72% | DO-7 | 0.01 | ********** |
| 98 | bukholm | p | 2000 | 93 | 93 | colon | IHC | survival | post surg. adj. | 60% | DO-1 | 0.1 | ********** |

| id | study | pa | year | size with data | size overall | site | method | endpoint | context | % with abnormal p53 | antibody | criterion | from exon |
|---|---|---|---|---|---|---|---|---|---|---|---|---|---|
| 97 | bukholm | p | 2000 | 93 | 93 | colon | IHC | metastases | post surg. adj. | 60% | DO-1 | 0.1 | ********** |
| 60 | caldes | p | 1998 | 72 | 72 | colorectal | mut | survival | post surg. adj. | 26% | ********** | ********** | 5 |
| 43 | caldes | p | 1998 | 72 | 72 | colorectal | IHC | survival | post surg. adj. | 56% | DO-7 | | ********** |
| 99 | cascinu | p | 2000 | 41 | 41 | colorectal | IHC | response | advanced / mets. | 66% | 53-12 | 0.2 | ********** |
| 48 | cascinu | p | 2002 | 79 | 79 | rectum | IHC | FFS | post surg. adj. | 46% | DO-7 | 0.1 | ********** |
| 100 | chen | p | 1997 | 70 | 70 | colorectal | IHC | FFS | post surg. adj. | 69% | Pab-1801 | 0.01 | ********** |
| 101 | chen | p | 1997 | 45 | 45 | colorectal | IHC | FFS | post surg. adj. | 69% | Pab-1801 | 0.01 | ********** |
| 102 | chen | p | 1997 | 25 | 25 | colorectal | IHC | FFS | post surg. adj. | 68% | Pab-1801 | 0.01 | ********** |
| 103 | clarke | p | 1999 | 134 | 134 | colorectal | IHC | survival | post surg. adj. | 58% | DO-7 | 0.01 | ********** |
| 257 | costa | p | 1997 | 104 | 104 | colorectal | IHC | FFS | advanced / mets. | 59% | Pab-1801 | 0.05 | ********** |
| 105 | desai | p | 1996 | 23 | 23 | rectum | IHC | FFS | local preop | 43% | Pab-1801 | | ********** |
| 104 | desai | p | 1996 | 20 | 23 | rectum | IHC | response | local preop | 43% | Pab-1801 | | ********** |
| 108 | diez | p | 1997 | 37 | 37 | rectum | IHC | FFS | local preop | 65% | CM-1 | 0.01 | ********** |
| 109 | diez | p | 1997 | 30 | 37 | rectum | IHC | FFS | local preop | 65% | CM-1 | 0.01 | ********** |
| 107 | diez | p | | 130 | 130 | rectum | IHC | FFS | post surg. adj. | 58% | CM-1 | 0.1 | ********** |
| 65 | diez | p | | 190 | 190 | colorectal | IHC | FFS | post surg. adj. | 53% | CM-1 | 0.1 | ********** |
| 69 | diez | p | 2000 | 174 | 174 | colorectal | IHC | FFS | post surg. adj. | 47% | CM-1 | 0.01 | ********** |
| 74 | diez | p | 1995 | 61 | 61 | colorectal | IHC | FFS | post surg. adj. | 44% | CM-1 | 0.01 | ********** |
| 106 | diez | p | | 60 | 60 | colon | IHC | FFS | post surg. adj. | 42% | CM-1 | 0.1 | ********** |
| 110 | dominguez | a | 1994 | 73 | 73 | colorectal | IHC | survival | post surg. adj. | 49% | DO-7 | 0.99 | ********** |
| 112 | edler | p | 2000 | 109 | 109 | rectum | IHC | survival | local preop | 69% | DO-7 | 0.05 | ********** |
| 111 | edler | p | 2000 | 109 | 109 | rectum | IHC | metastases | local preop | 69% | DO-7 | 0.05 | ********** |
| 114 | elsaleh | p | 2000 | 48 | 48 | rectum | mut | response | local preop | 35% | ********** | ********** | 5 |
| 115 | elsaleh | p | 2000 | 48 | 48 | rectum | other | response | local preop | 46% | ********** | ********** | |
| 15 | elsaleh | p | 1999 | 122 | 122 | rectum | mut | survival | post surg. adj. | 39% | ********** | ********** | 5 |
| 113 | elsaleh | p | 2000 | 48 | 48 | rectum | IHC | response | local preop | 40% | DO-7 | 0.05 | ********** |
| 17 | elsaleh | p | 1999 | 118 | 122 | rectum | IHC | survival | post surg. adj. | 39% | DO-7 | 0.05 | ********** |
| 116 | esposito | p | 2001 | 38 | 38 | rectum | IHC | response | local preop | 45% | Pab-1801 | 0.1 | ********** |
| 66 | etienne | p | 2002 | 97 | 97 | colorectal | mut | survival | advanced / mets. | 57% | ********** | ********** | 4 |
| 117 | fante | p | 1996 | 150 | 150 | colorectal | IHC | survival | post surg. adj. | 47% | Pab-1801 | 0.01 | ********** |
| 242 | finkelstein | p | 1996 | 204 | 204 | colorectal | mut | survival | post surg. adj. | 67% | ********** | ********** | 5 |
| 118 | flamini | p | 1996 | 94 | 94 | colorectal | IHC | FFS | post surg. adj. | 48% | DO-7 | 0.01 | ********** |

| id | study | pa | year | size with data | size overall | site | method | endpoint | context | % with abnormal p53 | antibody | criterion | from exon |
|---|---|---|---|---|---|---|---|---|---|---|---|---|---|
| 248 | fraczadi | p | 2003 | 20 | 20 | rectum | other | survival | local preop | | other | 99 | ********** |
| 120 | fu | p | 1998 | 46 | 46 | rectum | IHC | survival | local preop | 48% | DO-7 | 0.05 | ********** |
| 119 | fu | p | 1998 | 49 | 49 | rectum | IHC | response | local preop | 49% | DO-7 | 0.05 | ********** |
| 121 | gallego | p | 2000 | 126 | 126 | rectum | IHC | survival | local preop | 67% | DO-7 | 0.01 | ********** |
| 122 | gallego | p | 2000 | 126 | 126 | rectum | IHC | survival | post surg. adj. | 67% | DO-7 | 0.01 | ********** |
| 255 | garrity | p | 2004 | 365 | 1388 | colon | IHC | FFS | post surg. adj. | 76% | DO-7 | 0.99 | ********** |
| 254 | garrity | p | 2004 | 365 | 1388 | colon | IHC | survival | post surg. adj. | 76% | DO-7 | 0.99 | ********** |
| 123 | gervaz | p | 2001 | 122 | 122 | colorectal | mut | survival | post surg. adj. | 32% | ********** | ********** | 5 |
| 47 | gervaz | p | 2001 | 154 | 122 | colorectal | IHC | survival | post surg. adj. | 47% | DO-7 | 0.1 | ********** |
| 70 | gervaz | p | 2002 | 88 | 88 | colorectal | IHC | survival | post surg. adj. | 44% | DO-7 | 0.1 | ********** |
| 129 | giatromanc | p | 1999 | 106 | 106 | colorectal | IHC | survival | post surg. adj. | 44% | DO-7 | 0.1 | ********** |
| 126 | giatromanc | p | 2001 | 268 | 268 | colorectal | IHC | FFS | post surg. adj. | 46% | DO-7 | 0.05 | ********** |
| 128 | giatromanc | p | 1999 | 108 | 106 | colorectal | IHC | FFS | post surg. adj. | 44% | DO-7 | 0.1 | ********** |
| 127 | giatromanc | p | 2001 | 70 | 70 | rectum | IHC | FFS | post surg. adj. | 29% | DO-7 | 0.05 | ********** |
| 124 | giatromanc | p | 2001 | 268 | 268 | colorectal | IHC | local control | post surg. adj. | 46% | DO-7 | 0.05 | ********** |
| 125 | giatromanc | p | 2001 | 70 | 70 | rectum | IHC | local control | post surg. adj. | 29% | DO-7 | 0.05 | ********** |
| 131 | goh | p | 1999 | 130 | 130 | rectum | mut | survival | post surg. adj. | 55% | ********** | ********** | 4 |
| 132 | goh | p | 1999 | 198 | 198 | colon | mut | survival | post surg. adj. | 47% | ********** | ********** | 4 |
| 130 | goh | p | 1999 | 328 | 328 | colorectal | mut | metastases | post surg. adj. | 51% | ********** | ********** | 4 |
| 241 | gonzalez-a | p | 2004 | 77 | 96 | colorectal | mut | FFS | post surg. adj. | 39% | ********** | ********** | |
| 134 | grewal | p | 1995 | 66 | 66 | colorectal | IHC | survival | post surg. adj. | 52% | Pab-1801 | 0.01 | ********** |
| 133 | grewal | p | 1995 | 66 | 66 | colorectal | IHC | local control | post surg. adj. | 52% | Pab-1801 | 0.01 | ********** |
| 16 | gunther | p | 2002 | 115 | 115 | rectum | IHC | metastases | post surg. adj. | 58% | DO-1 | 0.1 | ********** |
| 135 | hallak | p | 1998 | 119 | 120 | colorectal | IHC | metastases | post surg. adj. | 55% | DO-1 | 0.99 | ********** |
| 51 | hamelin | p | 1994 | 85 | 85 | colorectal | mut | survival | post surg. adj. | 52% | ********** | ********** | 5 |
| 42 | hardinghan | p | 1998 | 100 | 100 | colorectal | mut | survival | post surg. adj. | 55% | ********** | ********** | 5 |
| 71 | haseba | p | 2003 | 73 | 79 | colorectal | mut | FFS | post surg. adj. | 71% | ********** | ********** | 5 |
| 136 | hayashi | p | 1995 | 61 | 61 | colorectal | mut | survival | post surg. adj. | 20% | ********** | ********** | 5 |
| 137 | heisterkam | p | 1999 | 45 | 45 | colorectal | IHC | FFS | advanced / mets. | 53% | DO-7 | 0.1 | ********** |
| 138 | hirano | p | 2000 | 71 | 71 | colorectal | IHC | metastases | post surg. adj. | 35% | CM-1 | 0.3 | ********** |
| 140 | hirvikoski | p | 1999 | 98 | 98 | rectum | mut | survival | post surg. adj. | 39% | ********** | ********** | 4 |
| 24 | hirvikoski | p | 1999 | 96 | 96 | rectum | IHC | survival | post surg. adj. | 54% | CM-1 | 0.2 | ********** |

| id | study | pa | year | size with data | size overall | site | method | endpoint | context | % with abnormal p53 | antibody | criterion | from exon |
|---|---|---|---|---|---|---|---|---|---|---|---|---|---|
| 139 | hirvikoski | p | 1999 | 97 | 97 | rectum | IHC | survival | post surg. adj. | 71% | DO-7 | 0.2 | ********** |
| 141 | ichikawa | p | 1996 | 15 | 15 | rectum | mut | response | local preop | 53% | ********** | ********** | 5 |
| 142 | ilyas | p | 1998 | 66 | 66 | colorectal | IHC | FFS | post surg. adj. | 56% | DO-7 | 0.1 | ********** |
| 68 | iniesta | p | 1998 | 37 | 61 | colorectal | mut | survival | post surg. adj. | 30% | ********** | ********** | 5 |
| 143 | jansson | p | 2001 | 75 | 75 | colorectal | mut | survival | post surg. adj. | 55% | ********** | ********** | 5 |
| 144 | jingwei | a | 1998 | 40 | 40 | colorectal | IHC | survival | post surg. adj. | 50% | DO-7 | 0.99 | ********** |
| 145 | kahlenberg | p | 2000 | 56 | 56 | colorectal | mut | survival | post surg. adj. | 50% | ********** | ********** | 5 |
| 22 | kahlenberg | p | 2000 | 56 | 56 | colorectal | mut | FFS | post surg. adj. | 50% | ********** | ********** | 5 |
| 149 | kandolier | p | 2002 | 64 | 64 | rectum | mut | survival | local preop | 45% | ********** | ********** | 2 |
| 148 | kandolier | p | 2002 | 64 | 64 | rectum | mut | local control | local preop | 45% | ********** | ********** | 2 |
| 147 | kandolier | p | 2002 | 64 | 64 | rectum | mut | response | local preop | 45% | ********** | ********** | 2 |
| 146 | kandolier | p | 2002 | 64 | 64 | rectum | mut | metastases | local preop | 45% | ********** | ********** | 2 |
| 151 | kang | p | 1997 | 163 | 163 | colorectal | IHC | survival | post surg. adj. | 42% | DO-7 | 0.1 | ********** |
| 150 | kang | p | 1997 | 101 | 101 | colorectal | IHC | FFS | post surg. adj. | 40% | DO-7 | 0.1 | ********** |
| 152 | kapiteijn | p | 2001 | 39 | 39 | rectum | IHC | survival | post surg. adj. | 64% | DO-7 | 0.25 | ********** |
| 153 | kapiteijn | p | 2001 | 34 | 34 | colon | IHC | survival | post surg. adj. | 29% | DO-7 | 0.25 | ********** |
| 154 | khan | p | 2001 | 27 | 27 | colorectal | mut | response | advanced / mets. | 78% | ********** | ********** | 4 |
| 155 | kim | p | 2001 | 23 | 23 | rectum | IHC | response | local preop | 70% | DO-7 | 0.1 | ********** |
| 156 | kim | p | 1995 | 387 | 471 | colorectal | IHC | survival | post surg. adj. | 45% | DO-7 | 0.01 | ********** |
| 10 | klump | p | 2003 | 125 | 125 | colon | IHC | survival | post surg. adj. | 46% | DO-1 | 0.05 | ********** |
| 11 | klump | p | 2003 | 133 | 133 | colon | IHC | survival | post surg. adj. | 41% | DO-1 | 0.05 | ********** |
| 12 | klump | p | 2003 | 113 | 113 | colon | IHC | survival | post surg. adj. | 39% | DO-1 | 0.05 | ********** |
| 158 | kotera | p | 1997 | 58 | 58 | rectum | IHC | survival | local preop | 31% | rsp53 | 0.01 | ********** |
| 157 | kotera | p | 1997 | 58 | 58 | rectum | IHC | local control | local preop | 31% | rsp53 | 0.01 | ********** |
| 34 | kressner | p | 1999 | 186 | 186 | colorectal | mut | survival | post surg. adj. | 53% | ********** | ********** | 1 |
| 21 | kressner | p | 1999 | 192 | 186 | colorectal | IHC | survival | local preop | 53% | DO-7 | | ********** |
| 160 | kressner | p | 1996 | 260 | 260 | colorectal | IHC | survival | post surg. adj. | 54% | DO-7 | 0.01 | ********** |
| 159 | kressner | p | 1996 | 294 | 294 | colorectal | IHC | FFS | post surg. adj. | 55% | DO-7 | 0.01 | ********** |
| 161 | lanza | p | 1996 | 141 | 204 | colorectal | IHC | survival | post surg. adj. | 61% | DO-7 | 0.05 | ********** |
| 7 | lashner | p | 2003 | 75 | 75 | colorectal | IHC | survival | post surg. adj. | 51% | DO-7 | 0.05 | ********** |
| 162 | lazaris | p | 1995 | 60 | 60 | colorectal | IHC | survival | post surg. adj. | 47% | Pab-1801 | 0.1 | ********** |
| 164 | leahy | p | 1996 | 66 | 66 | colorectal | mut | survival | post surg. adj. | 41% | ********** | ********** | 5 |

| id | study | pa | year | size with data | size overall | site | method | endpoint | context | % with abnormal p53 | antibody | criterion | from exon |
|---|---|---|---|---|---|---|---|---|---|---|---|---|---|
| 163 | leahy | p | 1996 | 70 | 66 | colorectal | IHC | survival | post surg. adj. | 47% | DO-7 | 0.1 | ********** |
| 244 | lenz | p | | 36 | 36 | colorectal | mut | survival | advanced / mets. | 72% | ********** | ********** | 4 |
| 79 | lenz | p | 1998 | 45 | 45 | colon | IHC | survival | post surg. adj. | 60% | DO-7 | 0.1 | ********** |
| 75 | lenz | p | 1998 | 45 | 45 | colon | IHC | FFS | post surg. adj. | 60% | DO-7 | 0.1 | ********** |
| 166 | liang | p | 1999 | 166 | 166 | rectum | mut | FFS | post surg. adj. | 54% | ********** | ********** | 2 |
| 165 | liang | p | 1999 | 0 | 166 | rectum | IHC | FFS | post surg. adj. | 50% | DO-1 | 0.1 | ********** |
| 168 | luna-perez | p | 1998 | 26 | 26 | rectum | IHC | response | local preop | 54% | Pab-1801 | 0.1 | ********** |
| 31 | maeda | p | 1997 | 101 | 101 | colorectal | IHC | FFS | post surg. adj. | 39% | DO-7 | 0.01 | ********** |
| 54 | manne | p | 1997 | 131 | 131 | colorectal | IHC | survival | post surg. adj. | 45% | 53-12 | 0.1 | ********** |
| 169 | manne | p | 1998 | 504 | 504 | colorectal | IHC | survival | post surg. adj. | 52% | 53-12 | 0.1 | ********** |
| 170 | morrin | p | 1994 | 52 | 52 | colorectal | IHC | survival | post surg. adj. | 62% | DO-7 | 0.99 | ********** |
| 171 | mulder | p | 1995 | 109 | 109 | colorectal | IHC | survival | post surg. adj. | 39% | DO-7 | 0.3 | ********** |
| 172 | nasierowsk | p | 2001 | 27 | 204 | rectum | IHC | response | local preop | 48% | DO-7 | 0.1 | ********** |
| 173 | nathanson | p | 1994 | 84 | 84 | colorectal | IHC | survival | post surg. adj. | 62% | Pab-1801 | 0.01 | ********** |
| 174 | nehls | p | 1999 | 100 | 100 | rectum | IHC | FFS | local preop | 55% | DO-1 | 0.05 | ********** |
| 58 | nitti | p | 1998 | 69 | 69 | colorectal | IHC | survival | advanced / mets. | 64% | DO-1 | 0.1 | ********** |
| 175 | offner | p | 1995 | 109 | 109 | colorectal | IHC | survival | post surg. adj. | 55% | CM-1 | 0.01 | ********** |
| 177 | ogiwara | p | 1994 | 39 | 39 | rectum | IHC | survival | post surg. adj. | 36% | DO-7 | 0.99 | ********** |
| 176 | ogiwara | p | 1994 | 39 | 39 | rectum | IHC | local control | post surg. adj. | 36% | DO-7 | 0.99 | ********** |
| 178 | okonkwo | p | 2001 | 25 | 25 | rectum | IHC | response | local preop | 40% | DO-7 | 0.1 | ********** |
| 180 | okubo | p | 2001 | 110 | 110 | colorectal | mut | survival | post surg. adj. | 37% | ********** | ********** | 2 |
| 179 | okubo | p | 2001 | 110 | 110 | colorectal | IHC | survival | post surg. adj. | 44% | DO-7 | 0.2 | ********** |
| 182 | paradiso | p | 2002 | 75 | 80 | colorectal | IHC | survival | advanced / mets. | 43% | Pab-1801 | 0.05 | ********** |
| 181 | paradiso | p | 2002 | 75 | 80 | colorectal | IHC | response | advanced / mets. | 43% | Pab-1801 | 0.05 | ********** |
| 23 | paradiso | p | 2000 | 108 | 108 | colorectal | IHC | response | advanced / mets. | 37% | Pab-1801 | 0.05 | ********** |
| 183 | pereira | p | 1997 | 80 | 80 | colorectal | IHC | survival | post surg. adj. | 30% | DO-7 | 0.1 | ********** |
| 184 | pocard | c | 1998 | 21 | 21 | colorectal | IHC | FFS | advanced / mets. | 62% | DO-7 | 0.05 | ********** |
| 186 | poller | p | 1997 | 250 | 250 | colorectal | IHC | survival | post surg. adj. | 61% | DO-1 | 0.01 | ********** |
| 185 | poller | p | 1997 | 250 | 250 | colorectal | IHC | local control | post surg. adj. | 61% | DO-1 | 0.01 | ********** |
| 187 | pricolo | p | 1996 | 70 | 70 | colon | mut | survival | post surg. adj. | 61% | ********** | ********** | 5 |
| 62 | pricolo | p | 1997 | 141 | 141 | colon | mut | survival | post surg. adj. | 61% | ********** | ********** | 5 |
| 190 | qiu | p | 2000 | 72 | 72 | rectum | IHC | survival | local preop | 69% | DO-7 | | ********** |

| id | study | pa | year | size with data | size overall | site | method | endpoint | context | % with abnormal p53 | antibody | criterion | from exon |
|---|---|---|---|---|---|---|---|---|---|---|---|---|---|
| 189 | qiu | p | 2000 | 72 | 72 | rectum | IHC | FFS | local preop | 69% | DO-7 | | ********** |
| 188 | qiu | p | 2000 | 72 | 72 | rectum | IHC | response | local preop | 69% | DO-7 | | ********** |
| 9 | rau | p | 2003 | 51 | 66 | rectum | mut | survival | local preop | 16% | ********** | ********** | 5 |
| 8 | rau | p | 2003 | 65 | 66 | rectum | IHC | survival | local preop | 43% | DO-7 | | ********** |
| 57 | rebischung | p | 2002 | 86 | 86 | rectum | mut | survival | local preop | 51% | ********** | ********** | 2 |
| 191 | rebischung | p | 2002 | 86 | 86 | rectum | mut | response | local preop | 51% | ********** | ********** | 2 |
| 193 | remvikos | p | 1992 | 78 | 78 | colorectal | IHC | survival | post surg. adj. | 67% | multiple | 0.99 | ********** |
| 192 | remvikos | p | 1992 | 78 | 78 | colorectal | IHC | FFS | post surg. adj. | 67% | multiple | 0.99 | ********** |
| 35 | rew | p | 1996 | 82 | 82 | colorectal | IHC | survival | post surg. adj. | 50% | Pab-1801 | 0.99 | ********** |
| 197 | rodel | p | 2002 | 39 | 44 | rectum | IHC | FFS | local preop | 45% | DO-7 | 0.1 | ********** |
| 198 | rodel | p | 2002 | 54 | 54 | rectum | IHC | FFS | local preop | 46% | DO-7 | 0.1 | ********** |
| 196 | rodel | p | 2002 | 54 | 54 | rectum | IHC | local control | local preop | 46% | DO-7 | 0.1 | ********** |
| 195 | rodel | p | 2002 | 42 | 44 | rectum | IHC | response | local preop | 45% | DO-7 | 0.1 | ********** |
| 194 | rodel | p | 2002 | 54 | 54 | rectum | IHC | metastases | local preop | 46% | DO-7 | 0.1 | ********** |
| 200 | rosty | p | 2001 | 49 | 56 | colorectal | mut | survival | advanced / mets. | 63% | ********** | ********** | 4 |
| 199 | rosty | p | 2001 | 56 | 56 | colorectal | mut | response | advanced / mets. | 63% | ********** | ********** | 4 |
| 52 | russo | p | 2002 | 160 | 160 | colorectal | mut | survival | post surg. adj. | 43% | ********** | ********** | 5 |
| 45 | russo | p | 2002 | 160 | 160 | colorectal | mut | FFS | post surg. adj. | 43% | ********** | ********** | 5 |
| 201 | russo | p | 1998 | 35 | 35 | colorectal | mut | survival | advanced / mets. | 43% | ********** | ********** | 5 |
| 202 | sakakura | p | 1998 | 28 | 28 | rectum | mut | response | local preop | 50% | ********** | ********** | 4 |
| 25 | samowitz | p | 2002 | 1462 | 1464 | colon | mut | survival | post surg. adj. | 45% | ********** | ********** | 5 |
| 2 | saw | p | 2003 | 60 | 78 | rectum | mut | local control | local preop | 55% | ********** | ********** | 5 |
| 5 | saw | p | 2003 | 60 | 78 | rectum | mut | response | local preop | 55% | ********** | ********** | 5 |
| 204 | saw | p | 2002 | 60 | 60 | colorectal | mut | survival | advanced / mets. | 58% | ********** | ********** | 5 |
| 3 | saw | p | 2003 | 60 | 78 | rectum | IHC | survival | local preop | 55% | DO-7 | 0.01 | ********** |
| 1 | saw | p | 2003 | 60 | 78 | rectum | IHC | local control | local preop | 55% | DO-7 | 0.01 | ********** |
| 4 | saw | p | 2003 | 60 | 78 | rectum | IHC | response | local preop | 55% | DO-7 | 0.01 | ********** |
| 203 | saw | p | 2002 | 63 | 63 | colorectal | IHC | survival | advanced / mets. | 44% | DO-7 | 0.05 | ********** |
| 40 | schelwies | p | 2002 | 76 | 59 | colorectal | mut | survival | post surg. adj. | 47% | ********** | ********** | 5 |
| 205 | schelwies | p | 2002 | 57 | 57 | colorectal | mut | survival | advanced / mets. | 32% | ********** | ********** | 5 |
| 206 | schwandne | p | 2002 | 160 | 160 | rectum | IHC | survival | post surg. adj. | 39% | DO-1 | 0.1 | ********** |
| 208 | schwandne | p | 2000 | 160 | 160 | rectum | IHC | survival | post surg. adj. | 39% | DO-1 | 0.1 | ********** |

| id | study | pa | year | size with data | size overall | site | method | endpoint | context | % with abnormal p53 | antibody | criterion | from exon |
|---|---|---|---|---|---|---|---|---|---|---|---|---|---|
| 73 | schwandne | p | 2002 | 160 | 160 | rectum | IHC | FFS | post surg. adj. | 39% | DO-1 | 0.1 | ********** |
| 207 | schwandne | p | 2000 | 160 | 160 | rectum | IHC | FFS | post surg. adj. | 39% | DO-1 | 0.1 | ********** |
| 210 | scott | p | 1991 | 52 | 52 | colorectal | IHC | survival | post surg. adj. | 42% | Pab421 | 0.7 | ********** |
| 209 | scott | p | 1998 | 22 | 22 | rectum | IHC | response | local preop | 59% | DO-7 | 0.33 | ********** |
| 213 | smith | p | 1996 | 100 | 100 | colorectal | mut | survival | post surg. adj. | 51% | ********** | ********** | |
| 211 | smith | p | 1996 | 100 | 100 | colorectal | IHC | survival | post surg. adj. | 62% | Pab-1801 | 0.01 | ********** |
| 212 | smith | p | 1996 | 100 | 100 | colorectal | IHC | survival | post surg. adj. | 76% | Pab240 | 0.01 | ********** |
| 246 | smith | p | 2003 | 24 | 24 | colorectal | IHC | survival | post surg. adj. | | DO-1 | 0.99 | ********** |
| 247 | smith | p | 2003 | 24 | 24 | colorectal | IHC | survival | post surg. adj. | | DO-1 | 0.1 | ********** |
| 18 | soong | p | 1997 | 541 | 541 | colorectal | mut | survival | post surg. adj. | 36% | ********** | ********** | 5 |
| 19 | soong | p | 2000 | 948 | 948 | colorectal | mut | survival | post surg. adj. | 38% | ********** | ********** | 4 |
| 14 | soong | p | 1997 | 541 | 541 | colorectal | IHC | survival | post surg. adj. | 30% | DO-7 | 0.05 | ********** |
| 214 | sory | p | 1997 | 50 | 50 | colorectal | IHC | metastases | post surg. adj. | 40% | rsp53 | 0.33 | ********** |
| 215 | spitz | p | 1997 | 42 | 42 | rectum | IHC | response | local preop | 55% | DO-1 | 0.1 | ********** |
| 217 | starzynska | p | 1997 | 102 | 102 | colorectal | IHC | survival | post surg. adj. | 46% | CM-1 | 0.01 | ********** |
| 219 | starzynska | p | 1992 | 80 | 107 | colorectal | IHC | survival | post surg. adj. | 46% | CM-1 | 0.01 | ********** |
| 216 | starzynska | p | 1997 | 102 | 102 | colorectal | IHC | FFS | post surg. adj. | 46% | CM-1 | 0.01 | ********** |
| 218 | starzynska | p | 1992 | 80 | 107 | colorectal | IHC | FFS | post surg. adj. | 46% | CM-1 | 0.01 | ********** |
| 13 | sturm | p | 1999 | 40 | 40 | colorectal | mut | survival | advanced / mets. | 35% | ********** | ********** | 8 |
| 220 | sturm | p | 1999 | 40 | 40 | colorectal | IHC | survival | advanced / mets. | 60% | DO-7 | 0.1 | ********** |
| 221 | subramania | p | 1995 | 56 | 56 | rectum | IHC | local control | post surg. adj. | 46% | CM-1 | 0.01 | ********** |
| 32 | sun | p | 1993 | 238 | 238 | colorectal | IHC | survival | post surg. adj. | 39% | CM-1 | 0.01 | ********** |
| 55 | sun | p | 1995 | 238 | 238 | colorectal | IHC | survival | post surg. adj. | | CM-1 | 0.01 | ********** |
| 222 | sun | p | 1997 | 256 | 256 | colorectal | IHC | survival | post surg. adj. | 23% | CM-1 | 0.05 | ********** |
| 223 | sun | p | 1996 | 104 | 293 | colorectal | IHC | survival | post surg. adj. | 38% | CM-1 | 0.01 | ********** |
| 224 | suzuki | p | 1994 | 67 | 67 | colorectal | IHC | FFS | post surg. adj. | 51% | Pab-1801 | 0.01 | ********** |
| 225 | tollenaar | p | 1998 | 238 | 238 | colorectal | IHC | survival | post surg. adj. | 40% | DO-7 | 0.3 | ********** |
| 243 | tomoda | p | 1995 | 144 | 144 | colorectal | IHC | FFS | post surg. adj. | 25% | CM-1 | 0.99 | ********** |
| 56 | tortola | p | 1999 | 132 | 151 | colorectal | mut | survival | post surg. adj. | 50% | ********** | ********** | 4 |
| 226 | tullo | p | 1999 | 40 | 40 | colorectal | mut | FFS | advanced / mets. | 45% | ********** | ********** | 5 |
| 227 | wang | a | 1998 | 40 | 40 | colorectal | IHC | survival | post surg. adj. | 65% | other | 0.99 | ********** |
| 228 | watanabe | p | 2002 | 78 | 78 | rectum | IHC | survival | local preop | 38% | DO-7 | 0.4 | ********** |

| id | study | pa | year | size with data | size overall | site | method | endpoint | context | % with abnormal p53 | antibody | criterion | from exon |
|---|---|---|---|---|---|---|---|---|---|---|---|---|---|
| 229 | wiggenraac | p | 1998 | 123 | 123 | rectum | IHC | survival | post surg. adj. | 48% | DO-7 | 0.1 | ********** |
| 36 | wiggenraac | p | 1998 | 123 | 123 | rectum | IHC | local control | post surg. adj. | 48% | DO-7 | 0.1 | ********** |
| 230 | yamaguchi | p | 1993 | 203 | 203 | colorectal | IHC | survival | post surg. adj. | 60% | Pab-1801 | 0.01 | ********** |
| 232 | yamaguchi | p | 1992 | 100 | 100 | colorectal | IHC | survival | post surg. adj. | 61% | Pab-1801 | 0.99 | ********** |
| 231 | yamaguchi | p | 1992 | 76 | 76 | colorectal | IHC | FFS | post surg. adj. | 55% | Pab-1801 | 0.99 | ********** |
| 235 | yamamura | p | 1999 | 144 | 144 | colorectal | IHC | survival | post surg. adj. | 50% | CM-1 | 0.1 | ********** |
| 233 | yamamura | p | 1999 | 144 | 144 | colorectal | IHC | FFS | post surg. adj. | 50% | CM-1 | 0.1 | ********** |
| 234 | yamamura | p | 1999 | 142 | 142 | colorectal | IHC | FFS | post surg. adj. | 50% | CM-1 | 0.2 | ********** |
| 236 | yang | p | 2001 | 35 | 35 | colorectal | mut | survival | advanced / mets. | 66% | ********** | ********** | 5 |
| 238 | yoshikawa | p | 2002 | 63 | 63 | rectum | IHC | local control | post surg. adj. | 49% | DO-7 | 0.01 | ********** |
| 237 | yoshikawa | p | 2002 | 63 | 63 | rectum | IHC | metastases | post surg. adj. | 51% | DO-7 | 0.01 | ********** |
| 72 | zeng | p | 1994 | 107 | 107 | colorectal | IHC | survival | post surg. adj. | 47% | Pab-1801 | 0.01 | ********** |
| 63 | zeng | p | 1994 | 107 | 107 | colorectal | IHC | FFS | post surg. adj. | 47% | Pab-1801 | 0.01 | ********** |
| 239 | zeng | p | 1994 | 107 | 107 | colorectal | IHC | local control | post surg. adj. | 47% | Pab-1801 | 0.01 | ********** |
| 240 | zhang | p | 1999 | 235 | 245 | colorectal | IHC | survival | post surg. adj. | 62% | multiple | 0.01 | ********** |
| 245 | zirbes | p | 2000 | 294 | 294 | colorectal | IHC | survival | post surg. adj. | 68% | other | 0.5 | ********** |

| study | pa | year | to exon | exons | time-point | median fup | design | Chemo | city region | country |
|---|---|---|---|---|---|---|---|---|---|---|
| adell | p | 1999 | ********** | ********** | 6 | 5.25 | retro. cohort | 0% | linkoping | sweden |
| adell | p | 1999 | ********** | ********** | 8 | 5.25 | retro. cohort | 0% | linkoping | sweden |
| adell | p | 1999 | ********** | ********** | 8 | 5.25 | retro. cohort | 0% | linkoping | sweden |
| adrover | p | 1999 | ********** | ********** | 5 | 5 | retro. cohort | 13% | madrid | spain |
| ahnen | p | 1998 | ********** | ********** | 14 | 8 | retro sel. | 47% | swog | usa |
| allegra | p | 2003 | ********** | ********** | 5 | 5 | retro sel. | 62% | nsabp | usa |
| allegra | p | 2002 | ********** | ********** | 6 | 5 | retro sel. | 68% | ncctg | usa |
| allegra | p | 2003 | ********** | ********** | 5 | 5 | retro sel. | 62% | nsabp | usa |
| aoki | p | 2002 | 8 | 4 | 8 | | retro sel. | . | tokyo | japan |
| auvinenen | p | 1994 | ********** | ********** | 7 | | retro. cohort | . | tampere | finland |
| backus | p | 2001 | ********** | ********** | 5 | | prosp.cohort | 100% | amsterdam | netherlands |
| backus | p | 2001 | ********** | ********** | | | prosp.cohort | 100% | amsterdam | netherlands |
| baretton | p | 1996 | ********** | ********** | 5 | | retro. unsel. | . | munich | germany |
| bartik | p | 1997 | ********** | ********** | 8 | | retro. cohort | . | linkoping | sweden |
| bell | p | 1993 | ********** | ********** | 5 | 2.8 | prosp.cohort | . | leeds | uk |
| belluco | p | 1996 | ********** | ********** | 3 | 1.85 | prosp.cohort | 100% | mski | usa |
| belluco | p | 1996 | ********** | ********** | | 1.85 | prosp.cohort | 100% | mski | usa |
| benhattar | p | 1996 | 8 | 4 | 5 | | prosp.cohort | 100% | lausanne | switzerland |
| berglund | p | 2002 | ********** | ********** | | | retro. cohort | 100% | uppsala | sweden |
| bhatavdeka | p | 1997 | ********** | ********** | 2 | 2.5 | retro sel. | . | ahmedaba | india |
| bleeker | p | 2001 | 8 | 4 | 5 | 4 | prosp.cohort | 100% | groningen | netherlands |
| borreson-d | p | 1998 | 8 | 4 | 8 | 4.7 | prosp.cohort | 0% | oslo | norway |
| bosari | p | 1995 | 8 | 4 | 7 | | retro. cohort | . | burlington | usa |
| bosari | p | 1994 | ********** | ********** | 7 | | retro. cohort | . | burlington | usa |
| bouzouren | p | 2000 | 8 | 4 | | 5.5 | retro. cohort | . | lausanne | switzerland |
| bouzouren | p | 2000 | ********** | ********** | 6 | 5.5 | retro. cohort | . | lausanne | switzerland |
| brett | p | 1996 | ********** | ********** | | | prosp.cohort | 100% | liverpool | uk |
| buglioni | p | 2001 | ********** | ********** | 5 | | retro. cohort | 38% | rome | italy |
| buglioni | p | 1999 | ********** | ********** | 5 | | retro. cohort | . | rome | italy |
| buglioni | p | 2001 | ********** | ********** | 5 | | retro. cohort | 38% | rome | italy |
| buglioni | p | 1999 | ********** | ********** | 5 | | retro. cohort | . | rome | italy |
| bukholm | p | 2000 | ********** | ********** | | 4 | retro. cohort | . | nordbyhag | norway |

| study | pa | year | to exon | exons | time- point | median fup | design | Chemo | city region | country |
|---|---|---|---|---|---|---|---|---|---|---|
| bukholm | p | 2000 | ********** | ********** | | 4 | retro. cohort | . | nordbyhag[illegible] | norway |
| caldes | p | 1998 | 9 | 5 | 3 | 4 | prosp.cohort | . | madrid | spain |
| caldes | p | 1998 | ********** | ********** | 3 | 4 | prosp.cohort | . | madrid | spain |
| cascinu | p | 2000 | ********** | ********** | | | retro. cohort | 100% | parma | italy |
| cascinu | p | 2002 | ********** | ********** | 4 | | retro. cohort | 100% | parma | italy |
| chen | p | 1997 | ********** | ********** | | 2 | retro. cohort | 14% | minnesota | usa |
| chen | p | 1997 | ********** | ********** | | 2 | retro. cohort | . | minnesota | usa |
| chen | p | 1997 | ********** | ********** | | 2 | retro. cohort | . | minnesota | usa |
| clarke | p | 1999 | ********** | ********** | 5 | 5 | retro. cohort | . | dublin | ireland |
| costa | p | 1997 | ********** | ********** | 4 | | prosp.cohort | 0% | milan | italy |
| desai | p | 1996 | ********** | ********** | | | retro. cohort | 22% | st louis | usa |
| desai | p | 1996 | ********** | ********** | | | retro. cohort | 22% | st louis | usa |
| diez | p | 1997 | ********** | ********** | | 1.8 | retro. cohort | 0% | madrid | spain |
| diez | p | 1997 | ********** | ********** | | 1.8 | retro. cohort | 81% | madrid | spain |
| diez | p | | ********** | ********** | 5 | 2.5 | retro. cohort | . | madrid | spain |
| diez | p | | ********** | ********** | 5 | 2.5 | retro. cohort | . | madrid | spain |
| diez | p | 2000 | ********** | ********** | | 2.3 | retro. cohort | . | madrid | spain |
| diez | p | 1995 | ********** | ********** | 5 | | retro. cohort | . | madrid | spain |
| diez | p | | ********** | ********** | 5 | 2.5 | retro. cohort | . | madrid | spain |
| dominguez | a | 1994 | ********** | ********** | | | retro. cohort | . | asturias | spain |
| edler | p | 2000 | ********** | ********** | | 8 | retro. cohort | 0% | stockholm | sweden |
| edler | p | 2000 | ********** | ********** | | 8 | retro. cohort | 0% | stockholm | sweden |
| elsaleh | p | 2000 | 8 | 4 | | | retro. cohort | 100% | nedlands | australia |
| elsaleh | p | 2000 | | 1 | | | retro. cohort | 100% | nedlands | australia |
| elsaleh | p | 1999 | 8 | 4 | 5 | 4.8 | retro. cohort | 29% | nedlands | australia |
| elsaleh | p | 2000 | ********** | ********** | | | retro. cohort | 100% | nedlands | australia |
| elsaleh | p | 1999 | ********** | ********** | 5 | 4.8 | retro. cohort | 29% | nedlands | australia |
| esposito | p | 2001 | ********** | ********** | | | prosp.cohort | 100% | padova | italy |
| etienne | p | 2002 | 9 | 6 | 4 | | prosp.cohort | 100% | nice | france |
| fante | p | 1996 | ********** | ********** | 5 | | retro. cohort | . | modena | italy |
| finkelstein | p | 1996 | 8 | 4 | 5 | 7 | retro. cohort | 100% | pittsbugh r[illegible] | usa |
| flamini | p | 1996 | ********** | ********** | 4 | 3 | retro. cohort | . | rome | italy |

| study | pa | year | to exon | exons | time-point | median fup | design | Chemo | city region | country |
|---|---|---|---|---|---|---|---|---|---|---|
| fraczadi | p | 2003 | ********** | ********** | 2 | 1.5 | retro. cohort | 100% | budapest | hungary |
| fu | p | 1998 | ********** | ********** | | 4.5 | retro. cohort | 0% | tokyo | japan |
| fu | p | 1998 | ********** | ********** | | 4.5 | retro. cohort | 0% | tokyo | japan |
| gallego | p | 2000 | ********** | ********** | 5 | 5 | retro. cohort | 0% | madrid | spain |
| gallego | p | 2000 | ********** | ********** | 5 | 5 | retro. cohort | 0% | madrid | spain |
| garrity | p | 2004 | ********** | ********** | 5 | 8 | retro. sel. | 0% | minnesota | usa |
| garrity | p | 2004 | ********** | ********** | 5 | 8 | retro. sel. | 0% | minnesota | usa |
| gervaz | p | 2001 | 8 | 4 | | 5.5 | retro. cohort | 0% | lausanne | switzerland |
| gervaz | p | 2001 | ********** | ********** | 6 | 5.5 | retro. cohort | 0% | lausanne | switzerland |
| gervaz | p | 2002 | ********** | ********** | 6 | 5.5 | retro. cohort | 0% | lausanne | switzerland |
| giatromanc | p | 1999 | ********** | ********** | 3 | 2.3 | retro. cohort | 48% | athens | greece |
| giatromanc | p | 2001 | ********** | ********** | | 2.6 | retro. cohort | 40% | athens | greece |
| giatromanc | p | 1999 | ********** | ********** | | 2.3 | retro. cohort | 48% | athens | greece |
| giatromanc | p | 2001 | ********** | ********** | | 2.6 | retro. cohort | . | athens | greece |
| giatromanc | p | 2001 | ********** | ********** | | 2.6 | retro. cohort | 40% | athens | greece |
| giatromanc | p | 2001 | ********** | ********** | | 2.6 | retro. cohort | . | athens | greece |
| goh | p | 1999 | 9 | 6 | | 3.5 | retro. cohort | . | singapore | singapore |
| goh | p | 1999 | 9 | 6 | | 3.5 | retro. cohort | . | singapore | singapore |
| goh | p | 1999 | 9 | 6 | | 3.5 | retro. cohort | . | singapore | singapore |
| gonzalez-a | p | 2004 | | | 5 | | retro. cohort | . | madrid | spain |
| grewal | p | 1995 | ********** | ********** | 7 | 3.2 | retro. cohort | 0% | new york | usa |
| grewal | p | 1995 | ********** | ********** | | 3.2 | retro. cohort | 0% | new york | usa |
| gunther | p | 2002 | ********** | ********** | | 7 | retro sel. | 0% | erlangen | germany |
| hallak | p | 1998 | ********** | ********** | | | retro. cohort | . | ulm | germany |
| hamelin | p | 1994 | 8 | 4 | 6 | 4 | retro. cohort | 0% | paris | france |
| hardinghan | p | 1998 | 8 | 4 | 5 | | retro. cohort | . | woodville | australia |
| haseba | p | 2003 | 9 | 5 | 4 | 2.75 | retro. cohort | 44% | nagasaki | japan |
| hayashi | p | 1995 | 8 | 4 | | | retro. cohort | . | tokyo | japan |
| heisterkam | p | 1999 | ********** | ********** | 5 | | retro. cohort | . | rotterdam | netherlands |
| hirano | p | 2000 | ********** | ********** | | 4.2 | retro. cohort | . | kanazawa | japan |
| hirvikoski | p | 1999 | 8 | 5 | | 2.8 | retro sel. | . | kuopio | finland |
| hirvikoski | p | 1999 | ********** | ********** | | 2.8 | retro sel. | . | kuopio | finland |

| study | pa | year | to exon | exons | time-point | median fup | design | Chemo | city region | country |
|---|---|---|---|---|---|---|---|---|---|---|
| hirvikoski | p | 1999 | ********** | ********** | | 2.8 | retro sel. | . | kuopio | finland |
| ichikawa | p | 1996 | 9 | 5 | | | retro sel. | 100% | kyoto | japan |
| ilyas | p | 1998 | ********** | ********** | | 6 | retro sel. | . | london | uk |
| iniesta | p | 1998 | 9 | 5 | 4 | | retro. cohort | . | madrid | spain |
| jansson | p | 2001 | 9 | 5 | | 10 | retro. cohort | . | linkoping | sweden |
| jingwei | a | 1998 | ********** | ********** | | | retro sel. | . | wuhan | china |
| kahlenberg | p | 2000 | 9 | 5 | 5 | | prosp.cohort | . | san antonic | usa |
| kahlenberg | p | 2000 | 9 | 5 | 5 | | prosp.cohort | . | san antonic | usa |
| kandolier | p | 2002 | 10 | 9 | 4 | | prosp.cohort | 23% | vienna | austria |
| kandolier | p | 2002 | 10 | 9 | | | prosp.cohort | 23% | vienna | austria |
| kandolier | p | 2002 | 10 | 9 | | | prosp.cohort | 23% | vienna | austria |
| kandolier | p | 2002 | 10 | 9 | | | prosp.cohort | 23% | vienna | austria |
| kang | p | 1997 | ********** | ********** | 5 | 5 | retro. cohort | . | osaka | japan |
| kang | p | 1997 | ********** | ********** | 5 | 5 | retro. cohort | . | osaka | japan |
| kapiteijn | p | 2001 | ********** | ********** | 4 | | retro sel. | . | leiden | netherlands |
| kapiteijn | p | 2001 | ********** | ********** | 4 | | retro sel. | . | leiden | netherlands |
| khan | p | 2001 | 9 | 6 | | 1 | prosp.cohort | 100% | london | uk |
| kim | p | 2001 | ********** | ********** | | | prosp.cohort | 100% | seoul | korea |
| kim | p | 1995 | ********** | ********** | 5 | | retro. cohort | . | seoul | korea |
| klump | p | 2003 | ********** | ********** | 8 | 3.9 | retro. cohort | 0% | dusseldorf | germany |
| klump | p | 2003 | ********** | ********** | 8 | 3.9 | retro. cohort | 0% | dusseldorf | germany |
| klump | p | 2003 | ********** | ********** | 8 | 3.9 | retro. cohort | 0% | dusseldorf | germany |
| kotera | p | 1997 | ********** | ********** | 8 | | retro. cohort | . | hyogo | japan |
| kotera | p | 1997 | ********** | ********** | 8 | | retro. cohort | . | hyogo | japan |
| kressner | p | 1999 | 11 | 11 | 8 | | prosp.cohort | 0% | uppsala | sweden |
| kressner | p | 1999 | ********** | ********** | 8 | | prosp.cohort | 0% | uppsala | sweden |
| kressner | p | 1996 | ********** | ********** | 8 | | retro. cohort | 0% | uppsala | sweden |
| kressner | p | 1996 | ********** | ********** | | | retro. cohort | 0% | uppsala | sweden |
| lanza | p | 1996 | ********** | ********** | 3 | 2.5 | retro. cohort | 24% | ferrara | italy |
| lashner | p | 2003 | ********** | ********** | 10 | | retro. cohort | . | cleveland | usa |
| lazaris | p | 1995 | ********** | ********** | | | retro. cohort | . | athens | greece |
| leahy | p | 1996 | 8 | 4 | 5 | | retro. cohort | 0% | dublin | ireland |

| study | pa | year | to exon | exons | time-point | median fup | design | Chemo | city region | country |
|---|---|---|---|---|---|---|---|---|---|---|
| leahy | p | 1996 | ********** | ********** | 5 | 10 | retro. cohort | 0% | dublin | ireland |
| lenz | p |  | 11 | 8 | 2 |  | prosp.cohort | 100% | los angeles | usa |
| lenz | p | 1998 | ********** | ********** | 1 |  | retro. cohort | 0% | los angeles | usa |
| lenz | p | 1998 | ********** | ********** |  |  | retro. cohort | 0% | los angeles | usa |
| liang | p | 1999 | 11 | 10 | 7 |  | retro. cohort | 100% | taipei | taiwan |
| liang | p | 1999 | ********** | ********** |  |  | retro. cohort | 100% | taipei | taiwan |
| luna-perez | p | 1998 | ********** | ********** |  |  | retro. cohort | 100% | mexico city | mexico |
| maeda | p | 1997 | ********** | ********** | 5 | 5 | retro. cohort | . | osaka | japan |
| manne | p | 1997 | ********** | ********** | 10 |  | retro sel. | . | birmingham | usa |
| manne | p | 1998 | ********** | ********** | 10 | 4 | retro sel. | . | birmingham | usa |
| morrin | p | 1994 | ********** | ********** | 5 | 5 | retro. cohort | . | limerick | ireland |
| mulder | p | 1995 | ********** | ********** | 10 |  | retro. cohort | . | leiden | netherlands |
| nasierowski | p | 2001 | ********** | ********** |  |  | retro. cohort | 0% | warsaw | poland |
| nathanson | p | 1994 | ********** | ********** | 7 |  | retro. cohort | 0% | detroit | usa |
| nehls | p | 1999 | ********** | ********** | 10 | 6 | retro. cohort | . | dusseldorf | germany |
| nitti | p | 1998 | ********** | ********** | 4 | 3 | prosp.cohort | 26% | padova | italy |
| offner | p | 1995 | ********** | ********** | 10 | 7.9 | retro. cohort | 0% | munster | germany |
| ogiwara | p | 1994 | ********** | ********** |  |  | retro. cohort | . | hamamatsu | japan |
| ogiwara | p | 1994 | ********** | ********** |  |  | retro. cohort | . | hamamatsu | japan |
| okonkwo | p | 2001 | ********** | ********** |  |  | retro. cohort | 100% | chicago | usa |
| okubo | p | 2001 | 11 | 10 | 5 |  | prosp.cohort | . | tokyo | japan |
| okubo | p | 2001 | ********** | ********** | 5 |  | prosp.cohort | . | tokyo | japan |
| paradiso | p | 2002 | ********** | ********** |  |  | retro. cohort | 94% | neuquen | argentina |
| paradiso | p | 2002 | ********** | ********** |  |  | retro. cohort | 94% | neuquen | argentina |
| paradiso | p | 2000 | ********** | ********** |  |  | retro. cohort | 100% | neuquen | argentina |
| pereira | p | 1997 | ********** | ********** |  | 5 | retro. cohort | 0% | lisbon | portugal |
| pocard | c | 1998 | ********** | ********** |  |  | retro. cohort | . | paris | france |
| poller | p | 1997 | ********** | ********** | 5 | 5 | retro. cohort | . | gloucester | uk |
| poller | p | 1997 | ********** | ********** |  | 5 | retro. cohort | . | gloucester | uk |
| pricolo | p | 1996 | 8 | 4 | 7 |  | retro. cohort | 100% | providence | usa |
| pricolo | p | 1997 | 8 | 4 | 7 |  | retro. cohort | 50% | providence | usa |
| qiu | p | 2000 | ********** | ********** |  |  | retro. cohort | 71% | minnesota | usa |

| study | pa | year | to exon | exons | time-point | median fup | design | Chemo | city region | country |
|---|---|---|---|---|---|---|---|---|---|---|
| qiu | p | 2000 | ********** | ********** | | | retro. cohort | 71% | minnesota | usa |
| qiu | p | 2000 | ********** | ********** | | | retro. cohort | 71% | minnesota | usa |
| rau | p | 2003 | 8 | 4 | 3 | | prosp.cohort | 100% | berlin | germany |
| rau | p | 2003 | ********** | ********** | 3 | | prosp.cohort | 100% | berlin | germany |
| rebischung | p | 2002 | 11 | 10 | 5 | 4 | prosp.cohort | 0% | lyon | france |
| rebischung | p | 2002 | 11 | 10 | | 4 | prosp.cohort | 0% | lyon | france |
| remvikos | p | 1992 | ********** | ********** | 5 | | retro. cohort | . | paris | france |
| remvikos | p | 1992 | ********** | ********** | 5 | | retro. cohort | . | paris | france |
| rew | p | 1996 | ********** | ********** | 4 | 2.5 | prosp.cohort | . | leicester | uk |
| rodel | p | 2002 | ********** | ********** | | | retro. cohort | 100% | erlangen | germany |
| rodel | p | 2002 | ********** | ********** | | 2.3 | retro. cohort | 100% | erlangen | germany |
| rodel | p | 2002 | ********** | ********** | | 2.3 | retro. cohort | 100% | erlangen | germany |
| rodel | p | 2002 | ********** | ********** | | | retro. cohort | 100% | erlangen | germany |
| rodel | p | 2002 | ********** | ********** | | 2.3 | retro. cohort | 100% | erlangen | germany |
| rosty | p | 2001 | 9 | 6 | 3 | | prosp.cohort | 100% | nice | france |
| rosty | p | 2001 | 9 | 6 | | | prosp.cohort | 100% | nice | france |
| russo | p | 2002 | 8 | 4 | 5 | 6 | prosp.cohort | 6% | palermo | italy |
| russo | p | 2002 | 8 | 4 | 5 | 6 | prosp.cohort | 6% | palermo | italy |
| russo | p | 1998 | 8 | 4 | | | prosp.cohort | . | palermo | italy |
| sakakura | p | 1998 | 8 | 5 | | | prosp.cohort | 100% | kyoto | japan |
| samowitz | p | 2002 | 8 | 4 | | 5 | retro. cohort | . | utah | usa |
| saw | p | 2003 | 8 | 4 | | 3.9 | retro. cohort | 45% | sydney | australia |
| saw | p | 2003 | 8 | 4 | | 3.9 | retro. cohort | 45% | sydney | australia |
| saw | p | 2002 | 8 | 4 | | | retro. cohort | . | sydney | australia |
| saw | p | 2003 | ********** | ********** | | 3.9 | retro. cohort | 45% | sydney | australia |
| saw | p | 2003 | ********** | ********** | | 3.9 | retro. cohort | 45% | sydney | australia |
| saw | p | 2003 | ********** | ********** | | 3.9 | retro. cohort | 45% | sydney | australia |
| saw | p | 2002 | ********** | ********** | | | retro. cohort | . | sydney | australia |
| schelwies | p | 2002 | 8 | 4 | 8 | 5 | retro. cohort | 5% | berlin | germany |
| schelwies | p | 2002 | 8 | 4 | 8 | 5 | retro. cohort | 5% | berlin | germany |
| schwandne | p | 2002 | ********** | ********** | | 3.2 | retro. cohort | 57% | lubeck | germany |
| schwandne | p | 2000 | ********** | ********** | 6 | 3.2 | retro. cohort | 57% | lubeck | germany |

| study | pa | year | to exon | exons | time- point | median fup | design | Chemo | city region | country |
|---|---|---|---|---|---|---|---|---|---|---|
| schwandne | p | 2002 | ********** | ********** | | 3.2 | retro. cohort | 57% | lubeck | germany |
| schwandne | p | 2000 | ********** | ********** | | 3.2 | retro. cohort | 57% | lubeck | germany |
| scott | p | 1991 | ********** | ********** | 3 | | prosp.cohort | . | leeds | uk |
| scott | p | 1998 | ********** | ********** | | | retro. cohort | 100% | stoke | uk |
| smith | p | 1996 | | 1 | 4 | 2 | prosp.cohort | 0% | singapore | singapore |
| smith | p | 1996 | ********** | ********** | 4 | 2 | prosp.cohort | 0% | singapore | singapore |
| smith | p | 1996 | ********** | ********** | 4 | 2 | prosp.cohort | . | singapore | singapore |
| smith | p | 2003 | ********** | ********** | 5 | 4 | retro. cohort | . | new york | usa |
| smith | p | 2003 | ********** | ********** | 5 | 4 | retro. cohort | . | new york | usa |
| soong | p | 1997 | 8 | 4 | 7 | 3 | retro. cohort | 5% | nedlands | australia |
| soong | p | 2000 | 1 | -2 | | | retro. cohort | 17% | nedlands | australia |
| soong | p | 1997 | ********** | ********** | 7 | 3 | retro. cohort | 5% | nedlands | australia |
| sory | p | 1997 | ********** | ********** | | | retro. cohort | . | kanazawa | japan |
| spitz | p | 1997 | ********** | ********** | | | retro. cohort | 100% | houston | usa |
| starzynska | p | 1997 | ********** | ********** | 5 | 5.5 | retro. cohort | 3% | mancheste | uk |
| starzynska | p | 1992 | ********** | ********** | | 1 | retro. cohort | . | mancheste | uk |
| starzynska | p | 1997 | ********** | ********** | 5 | 5.5 | retro. cohort | 3% | mancheste | uk |
| starzynska | p | 1992 | ********** | ********** | | 1 | retro. cohort | . | mancheste | uk |
| sturm | p | 1999 | | -7 | 6 | | retro. cohort | 73% | berlin | germany |
| sturm | p | 1999 | ********** | ********** | 6 | | retro. cohort | 73% | berlin | germany |
| subramania | p | 1995 | ********** | ********** | | 2.3 | retro. cohort | . | hobart | australia |
| sun | p | 1993 | ********** | ********** | 8 | | retro. cohort | . | linkoping | sweden |
| sun | p | 1995 | ********** | ********** | 8 | | retro. cohort | . | linkoping | sweden |
| sun | p | 1997 | ********** | ********** | 10 | 10 | retro. cohort | . | linkoping | sweden |
| sun | p | 1996 | ********** | ********** | 8 | | retro. cohort | . | linkoping | sweden |
| suzuki | p | 1994 | ********** | ********** | 3 | 2 | prosp.cohort | . | edobashi | japan |
| tollenaar | p | 1998 | ********** | ********** | 7 | 2 | retro. cohort | 0% | leiden | netherlands |
| tomoda | p | 1995 | ********** | ********** | 2 | 1.5 | retro. cohort | 0% | fukuoka | japan |
| tortola | p | 1999 | 9 | 6 | 5 | 4.3 | retro. cohort | 21% | barcelona | spain |
| tullo | p | 1999 | 9 | 5 | | 2.3 | prosp.cohort | . | bari | italy |
| wang | a | 1998 | ********** | ********** | | | retro. cohort | . | | china |
| watanabe | p | 2002 | ********** | ********** | 5 | | retro. cohort | 0% | tokyo | japan |

| study | pa | year | to exon | exons | time-point | median fup | design | Chemo | city region | country |
|---|---|---|---|---|---|---|---|---|---|---|
| wiggenraac | p | 1998 | ********** | ********** | 6 | 3.3 | retro. cohort | 0% | leiden | netherlands |
| wiggenraac | p | 1998 | ********** | ********** | 6 | 3.3 | retro. cohort | 0% | leiden | netherlands |
| yamaguchi | p | 1993 | ********** | ********** | 10 | | retro. cohort | . | kanazawa | japan |
| yamaguchi | p | 1992 | ********** | ********** | 4 | | retro. cohort | . | kanazawa | japan |
| yamaguchi | p | 1992 | ********** | ********** | | | retro. cohort | . | kanazawa | japan |
| yamamura | p | 1999 | ********** | ********** | 5 | | retro. cohort | 100% | kawasaki | japan |
| yamamura | p | 1999 | ********** | ********** | 5 | | retro. cohort | 100% | kawasaki | japan |
| yamamura | p | 1999 | ********** | ********** | | | retro. cohort | . | kawasaki | japan |
| yang | p | 2001 | 9 | 5 | 5 | | prosp.cohort | 0% | goteborg | sweden |
| yoshikawa | p | 2002 | ********** | ********** | | | retro. cohort | . | tsu | japan |
| yoshikawa | p | 2002 | ********** | ********** | | | retro. cohort | . | tsu | japan |
| zeng | p | 1994 | ********** | ********** | 6 | 5 | retro. cohort | 19% | new york | usa |
| zeng | p | 1994 | ********** | ********** | 6 | 5 | retro. cohort | 19% | new york | usa |
| zeng | p | 1994 | ********** | ********** | 6 | 5 | retro. cohort | 19% | new york | usa |
| zhang | p | 1999 | ********** | ********** | 8 | | retro. cohort | . | linkoping | sweden |
| zirbes | p | 2000 | ********** | ********** | 10 | 4.7 | retro. cohort | . | cologne | germany |
