# Supplementary material for: P53 abnormalities and outcomes in colorectal cancer: a systematic review
Source: Br J Cancer. 2005 Jan 25;92(3):434–44. doi: 10.1038/sj.bjc.6602358 (PMC2362083; doi:10.1038/sj.bjc.6602358)
Supplement: Supplementary File 2 [file 92-6602358x2.pdf]

Studies included in meta-analysis of effects of abnormal p53 upon outcome in colorectal cancer

Adell, G., Sun, X.F., Stal, O., Klintenberg, C., Sjodahl, R. & Nordenskjold, B. (1999). p53 status: an indicator for the effect of preoperative radiotherapy of rectal cancer. *Radiotherapy & Oncology.*, **51,** 169-74.

Adrover, E., Maestro, M.L., Sanz-Casla, M.T., Del Barco, V., Cerdan, J., Fernandez, C. & Balibrea, J.L. (1999). Expression of high p53 levels in colorectal cancer: A favourable prognostic factor. *British Journal of Cancer.*, **81,** 122-126.

Ahnen, D.J., Feigl, P., Quan, G., Fenoglio-Preiser, C., Lovato, L.C., Bunn, P.A., Jr., Stemmerman, G., Wells, J.D., Macdonald, J.S. & Meyskens, F.L., Jr. (1998). Ki-ras mutation and p53 overexpression predict the clinical behavior of colorectal cancer: A Southwest Oncology Group study. *Cancer Research.*, **58,** 1149-1158.

Allegra, C.J., Paik, S., Colangelo, L.H., Parr, A.L., Kirsch, I., Kim, G., Klein, P., Johnston, P.G., Wolmark, N. & Wieand, H.S. (2003). Prognostic value of thymidylate synthase, Ki-67, and p53 in patients with Dukes' B and C colon cancer: a National Cancer Institute-National Surgical Adjuvant Breast and Bowel Project collaborative study. *Journal of Clinical Oncology*, **21,** 241-50.

Allegra, C.J., Parr, A.L., Wold, L.E., Mahoney, M.R., Sargent, D.J., Johnston, P., Klein, P., Behan, K., O'Connell, M.J., Levitt, R., Kugler, J.W., Tria Tirona, M. & Goldberg, R.M. (2002). Investigation of the prognostic and predictive value of thymidylate synthase, p53, and Ki-67 in patients with locally advanced colon cancer. *Journal of Clinical Oncology.*, **20,** 1735-43.

Aoki, T., Katsumata, K., Tsuchida, A., Tomioka, H. & Koyanagi, Y. (2002). Correlation between malignancy grade and p53 gene in relation to thymidine phosphorylase activity in colorectal cancer patients. *Oncology Reports*, **9,** 1267-71.

Auvinen, A., Isola, J., Visakorpi, T., Koivula, T., Virtanen, S. & Hakama, M. (1994). Overexpression of p53 and long-term survival in colon carcinoma. *British Journal of Cancer.*, **70,** 293-296.

Backus, H.H., van Riel, J.M., van Groeningen, C.J., Vos, W., Dukers, D.F., Bloemena, E., Wouters, D., Pinedo, H.M. & Peters, G.J. (2001). Rb, mcl-1 and p53 expression correlate with clinical outcome in patients with liver metastases from colorectal cancer. *Annals of Oncology.*, **12,** 779-85.

Baretton, G.B., Vogt, M., Muller, C., Diebold, J., Schneiderbanger, K., Schmidt, M. & Lohrs, U. (1996). Prognostic significance of p53 expression, chromosome 17 copy number, and DNA ploidy in non-metastasized colorectal carcinomas (Stages IB and II). *Scandinavian Journal of Gastroenterology.*, **31,** 481-489.

Bartik, Z., Nordenskjold, B. & Sun, X.F. (1997). p53 Overexpression as a prognostic factor in patients with Dukes B colorectal adenocarcinoma. *International Journal of Oncology.*, **11,** 1019-1023.

Bell, S.M., Scott, N., Cross, D., Sagar, P., Lewis, F.A., Blair, G.E., Taylor, G.R., Dixon, M.F. & Quirke, P. (1993). Prognostic value of p53 overexpression and c-Ki-ras gene mutations in colorectal cancer. *Gastroenterology.*, **104,** 57-64.

Belluco, C., Guillem, J.G., Kemeny, N., Huang, Y., Klimstra, D., Berger, M.F. & Cohen, A.M. (1996). p53 nuclear protein overexpression in colorectal cancer: a dominant predictor of survival in patients with advanced hepatic metastases. *Journal of Clinical Oncology : Official Journal of the American Society of Clinical Oncology*, **14,** 2696-701.

Benhattar, J., Cerottini, J.P., Saraga, E., Metthez, G. & Givel, J.C. (1996). p53 mutations as a possible predictor of response to chemotherapy in metastatic colorectal carcinomas. *International Journal of Cancer.*, **69,** 190-2.

Berglund, A., Edler, D., Molin, D., Nordlinder, H., Graf, W. & Glimelius, B. (2002). Thymidylate synthase and p53 expression in primary tumor do not predict chemotherapy outcome in metastatic colorectal carcinoma. *Anticancer Research*, **22,** 3653-9.

Bhatavdekar, J.M., Patel, D.D., Ghosh, N., Chikhlikar, P.R., Trivedi, T.I., Suthar, T.P., Doctor, S.S., Shah, N.G. & Balar, D.B. (1997). Coexpression of Bcl-2, c-Myc, and p53 oncoproteins as prognostic discriminants in patients with colorectal carcinoma. *Diseases of the Colon and Rectum*, **40,** 785-790.

Bleeker, W.A., Hayes, V.M., Karrenbeld, A., Hofstra, R.M., Verlind, E., Hermans, J., Poppema, S., Buys, C.H. & Plukker, J.T. (2001). Prognostic significance of K-ras and TP53 mutations in the role of adjuvant chemotherapy on survival in patients with Dukes C colon cancer. *Diseases of the Colon & Rectum.*, **44,** 358-63.

Borresen-Dale, A.L., Lothe, R.A., Meling, G.I., Hainaut, P., Rognum, T.O. & Skovlund, E. (1998). TP53 and long-term prognosis in colorectal cancer: Mutations in the L3 zinc-binding domain predict poor survival. *Clinical Cancer Research.*, **4,** 203-210.

Bosari, S., Viale, G., Bossi, P., Maggioni, M., Coggi, G., Murray, J.J. & Lee, A.K.C. (1994). Cytoplasmic accumulation of p53 protein: An independent prognostic indicator in colorectal adenocarcinomas. *Journal of the National Cancer Institute*, **86,** 681-687.

Bosari, S., Viale, G., Roncalli, M., Graziani, D., Borsani, G., Lee, A.K.C. & Coggi, G. (1995). p53 Gene mutations, p53 protein accumulation and compartmentalization in colorectal adenocarcinoma. *American Journal of Pathology.*, **147,** 790-798.

Bouzourene, H., Gervaz, P., Cerottini, J.P., Benhattar, J., Chaubert, P., Saraga, E., Pampallona, S., Bosman, F.T. & Givel, J.C. (2000). p53 and Ki-ras as prognostic factors for Dukes' stage B colorectal cancer. *European Journal of Cancer.*, **36,** 1008-1015.

Brett, M.C., Pickard, M., Green, B., Howel-Evans, A., Smith, D., Kinsella, A. & Poston, G. (1996). p53 protein overexpression and response to biomodulated 5-fluorouracil chemotherapy in patients with advanced colorectal cancer. *European Journal of Surgical Oncology.*, **22,** 182-185.

Buglioni, S., D'Agnano, I., Cosimelli, M., Vasselli, S., D'Angelo, C., Tedesco, M., Zupi, G. & Mottolese, M. (1999). Evaluation of multiple bio-pathological factors in colorectal adenocarcinomas: Independent prognostic role of p53 and Bcl-2. *International Journal of Cancer.*, **84,** 545-552.

Buglioni, S., D'Agnano, I., Vasselli, S., Donnorso, R.P., D'Angelo, C., Brenna, A., Benevolo, M., Cosimelli, M., Zupi, G. & Mottolese, M. (2001). p53 nuclear accumulation and multiploidy are adverse prognostic factors in surgically resected stage II colorectal cancers independent of fluorouracil-based adjuvant therapy. *American Journal of Clinical Pathology.*, **116,** 360-368.

Bukholm, I.K. & Nesland, J.M. (2000). Protein expression of p53, p21 (WAF1/CIP1), bcl-2, Bax, cyclin D1 and pRb in human colon carcinomas. *Virchows Archiv.*, **436,** 224-228.

Caldes, T., Iniesta, P., Vega, F.J., De Juan, C., Lopez, J.A., Diaz-Rubio, E., Fernandez, C., Cerdan, J., Balibrea, J.L. & Benito, M. (1998). Comparative survival analysis of p53 gene mutations and protein accumulation in colorectal cancer. *Oncology.*, **55,** 249-257.

Cascinu, S., Catalano, V., Aschele, C., Barni, S., Debernardis, D., Gallo, L., Bandelloni, R., Staccioli, M.P., Baldelli, A.M., Brenna, A., Valenti, A., Muretto, P. & Catalano, G. (2000). Immunohistochemical determination of p53 protein does not predict clinical response in advanced colorectal cancer with low thymidylate synthase expression receiving a bolus 5-fluorouracil-leucovorin combination. *Annals of Oncology : Official Journal of the European Society For Medical Oncology / Esmo*, **11,** 1053-6.

Cascinu, S., Graziano, F., Catalano, V., Staccioli, M.P., Rossi, M.C., Baldelli, A.M., Barni, S., Brenna, A., Secondino, S., Muretto, P. & Catalano, G. (2002). An analysis of p53, BAX and vascular endothelial growth factor expression in node-positive rectal cancer. Relationships with tumour recurrence and event-free survival of patients treated with adjuvant chemoradiation. *British Journal of Cancer.*, **86,** 744-749.

Chen, W.S., Chen, J.Y., Liu, J.M., Lin, W.C., King, K.L., Whang-Peng, J. & Yang, W.K. (1997). Microsatellite instability in sporadic-colon-cancer patients with and without liver metastases. *International Journal of Cancer.*, **74,** 470-474.

Clarke, G., Ryan, E., Crowe, J., O'Keane, J.C. & MacMathuna, P. (1999). Immunohistochemical detection of mutant p53 protein in regional lymph nodes is associated with adverse outcome in stage II colorectal cancer. *European Journal of Histochemistry.*, **43,** 311-6.

Clausen, O.P., Lothe, R.A., Borresen_Dale, A.L., De_Angelis, P., Chen, Y., Rognum, T.O. & Meling, G.I. (1998). Association of p53 accumulation with TP53 mutations, loss of heterozygosity at 17p13, and DNA ploidy status in 273 colorectal carcinomas. *Diagnostic Molecular Pathology : the American Journal of Surgical Pathology, Part B*, **7,** 215-23.

Costa, A., Doci, R., Mochen, C., Bignami, P., Faranda, A., Gennari, L. & Silvestrini, R. (1997). Cell proliferation-related markers in colorectal liver metastases: correlation with patient prognosis. *J Clin Oncol*, **15,** 2008-2014.

Cripps, K.J., Purdie, C.A., Carder, P.J., White, S., Komine, K., Bird, C.C. & Wyllie, A.H. (1994). A study of stabilisation of p53 protein versus point mutation in colorectal carcinoma. *Oncogene*, **9,** 2739-43.

Cunningham, J., Lust, J.A., Schaid, D.J., Bren, G.D., Carpenter, H.A., Rizza, E., Kovach, J.S. & Thibodeau, S.N. (1992). Expression of p53 and 17p allelic loss in colorectal carcinoma. *Cancer Research*, **52,** 1974-80.

Darmon, E., Cleary, K.R. & Wargovich, M.J. (1994). Immunohistochemical analysis of p53 overexpression in human colonic tumors. *Cancer Detection and Prevention*, **18,** 187-95.

Desai, G.R., Myerson, R.J., Higashikubo, R., Birnbaum, E., Fleshman, J., Fry, R., Kodner, I., Kucik, N., Lacey, D. & Ribeiro, M. (1996). Carcinoma of the rectum. Possible cellular predictors of metastatic potential and response to radiation therapy. *Diseases of the Colon and Rectum*, **39,** 1090-6.

Diez, M., Camunas, J., Enriquez, J.M., Gonzalez, A., Torabuela, E., Gutierrez, A., Ratia, T., Muguerza, J.M., Martin, A., Ruiz, A. & Granell, J. (1996). [A comparative study on the prognostic value of the nuclear expression of protein p53 vis-a-vis histopathology in colorectal cancer]. *Anales de Medicina Interna.*, **13,** 222-6.

Diez, M., Enriquez, J.M., Camunas, J., Gonzalez, A., Gutierrez, A., Muguerza, J.M., Ruiz, A. & Granell, J. (1995). Prediction of recurrence in B-C stages of colorectal cancer by p53 nuclear overexpression in comparison with standard pathological features. *European Journal of Surgical Oncology.*, **21,** 635-639.

Diez, M., Medrano, M., Muguerza, J.M., Ramos, P., Hernandez, P., Villeta, R., Martin, A., Noguerales, F., Ruiz, A. & Granell, J. (2000a). Influence of tumor localization on the prognostic value of p53 protein in colorectal adenocarcinomas. *Anticancer Research.*, **20,** 3907-3912.

Diez, M., Pollan, M., Muguerza, J.M., Gaspar, M.J., Duce, A.M., Alvarez, M.J., Ratia, T., Hernandez, P., Ruiz, A. & Granell, J. (2000b). Time-dependency of the prognostic effect of carcinoembryonic antigen and p53 protein in colorectal adenocarcinoma. *Cancer.*, **88,** 35-41.

Dominguez Iglesias, F., Riera Velasco, J.R., Junco Petrement, P., Tojo Ramallo, P. & Diaz-Faes Cervero, M. (1994). [Effect of overexpression of P53 protein in colorectal carcinoma on short-term prognosis]. *Revista Espanola de Enfermedades Digestivas.*, **86,** 796-802.

Edler, D., Kressner, U., Ragnhammar, P., Johnston, P.G., Magnusson, I., Glimelius, B., Pahlman, L., Lindmark, G. & Blomgren, H. (2000). Immunohistochemically detected thymidylate synthase in colorectal cancer: An independent prognostic factor of survival. *Clinical Cancer Research.*, **6,** 488-492.

Elsaleh, H., Powell, B., Soontrapornchai, P., Joseph, D., Goria, F., Spry, N. & Iacopetta, B. (2000a). p53 gene mutation, microsatellite instability and adjuvant chemotherapy: Impact on survival of 388 patients with Dukes' C colon carcinoma. *Oncology.*, **58,** 52-59.

Elsaleh, H., Robbins, P., Joseph, D., Powell, B., Grieu, F., Menso, L. & Iacopetta, B. (2000b). Can p53 alterations be used to predict tumour response to pre-operative chemo-radiotherapy in locally advanced rectal cancer? *Radiotherapy & Oncology*, **56,** 239-44.

Elsaleh, H., Soontrapornchai, P., Grieu, F., Joseph, D. & Iacopetta, B. (1999). P53 alterations have no prognostic or predictive significance in Dukes' C rectal carcinomas. *International Journal of Oncology.*, **15,** 1239-43.

Esposito, G., Pucciarelli, S., Alaggio, R., Giacomelli, L., Marchiori, E., Iaderosa, G.A., Friso, M.L., Toppan, P., Chieco-Bianchi, L. & Lise, M. (2001). P27kip1 expression is associated with tumor response to preoperative chemoradiotherapy in rectal cancer. *Annals of Surgical Oncology.*, **8,** 311-8.

Etienne, M.C., Chazal, M., Laurent-Puig, P., Magne, N., Rosty, C., Formento, J.L., Francoual, M., Formento, P., Renee, N., Chamorey, E., Bourgeon, A., Seitz, J.F., Delpero, J.R., Letoublon, C., Pezet, D. & Milano, G. (2002). Prognostic value of tumoral thymidylate synthase and p53 in metastatic colorectal cancer patients receiving fluorouracil-based chemotherapy: Phenotypic and genotypic analyses. *Journal of Clinical Oncology.*, **20,** 2832-2843.

Fante, R., Di Gregorio, C., Losi, L., Roncucci, L. & Ponz de Leon, M. (1996). Clinico-pathological correlation and prognostic significance of nuclear p53 protein in colorectal cancer. Colorectal Cancer Study Group of the University and Health Care District of Modena. *Italian Journal of Gastroenterology.*, **28,** 205-10.

Flamini, G., Curigliano, G., Ratto, C., Astone, A., Ferretti, G., Nucera, P., Sofo, L., Sgambato, A., Boninsegna, A., Crucitti, F. & Cittadini, A. (1996). Prognostic significance of cytoplasmic p53 overexpression in colorectal cancer. An immunohistochemical analysis. *European Journal of Cancer Part A*, **32,** 802-806.

Fu, C.G., Tominaga, O., Nagawa, H., Nita, M.E., Masaki, T., Ishimaru, G., Higuchi, Y., Tsuruo, T. & Muto, T. (1998). Role of p53 and p21/WAF1 detection in patient selection for preoperative radiotherapy in rectal cancer patients. *Diseases of the Colon and Rectum*, **41,** 68-74.

Gafa, R., Maestri, I., Matteuzzi, M., Santini, A., Ferretti, S., Cavazzini, L. & Lanza, G. (2000). Sporadic colorectal adenocarcinomas with high-frequency microsatellite instability: Pathobiologic features, hMLH1 and hMSH2 expression, and clinical outcome. *Cancer*, **89,** 2025-2037.

Gallego, M.G., Acenero, M.J., Ortega, S., Delgado, A.A. & Cantero, J.L. (2000). Prognostic influence of p53 nuclear overexpression in colorectal carcinoma. *Diseases of the Colon & Rectum.*, **43,** 971-5.

Garrity, M.M., Burgart, L.J., Mahoney, M.R., Windschitl, H.E., Salim, M., Wiesenfeld, M., Krook, J.E., Michalak, J.C., Goldberg, R.M., O'Connell, M.J., Furth, A.F., Sargent, D.J., Murphy, L.M., Hill, E., Riehle, D.L., Meyers, C.H., Witzig, T.E. & North Central Cancer Treatment, G. (2004). Prognostic value of proliferation, apoptosis, defective DNA mismatch repair, and p53 overexpression in patients with resected Dukes' B2 or C colon cancer: a North Central Cancer Treatment Group Study. *Journal of Clinical Oncology*, **22,** 1572-82.

Gervaz, P., Cerottini, J.P., Bouzourene, H., Hahnloser, D., Doan, C.L., Benhattar, J., Chaubert, P., Secic, M., Gillet, M. & Carethers, J.M. (2002). Comparison of microsatellite instability and chromosomal instability in predicting survival of patients with T3N0 colorectal cancer. *Surgery.*, **131,** 190-197.

Gervaz, P., Efron, J., Poza, A.A., Chun, S.W., Pham Thu-Thao, T., Woodhouse, S., Wexner, S.D. & Carethers, J.M. (2001). Loss of heterozygosity and HIV infection in patients with anal squamous-cell carcinoma. *Diseases of the Colon & Rectum.*, **44,** 1503-1508.

Giatromanolaki, A., Sivridis, E., Stathopoulos, G.P., Fountzilas, G., Kalofonos, H.P., Tsamandas, A., Vrettou, E., Scopa, C., Polychronidis, A., Simopoulos, K. & Koukourakis, M.I. (2001). Bax protein expression in colorectal cancer: Association with p53, bcl-2 and patterns of relapse. *Anticancer Research.*, **21,** 253-260.

Giatromanolaki, A., Stathopoulos, G.P., Tsiobanou, E., Papadimitriou, C., Georgoulias, V., Gatter, K.C., Harris, A.L. & Koukourakis, M.I. (1999). Combined role of tumor angiogenesis, bcl-2, and p53 expression in the prognosis of patients with colorectal carcinoma. *Cancer.*, **86,** 1421-1430.

Goh, H.S., Elnatan, J., Low, C.H. & Smith, D.R. (1999). p53 point mutation and survival in colorectal cancer patients: effect of disease dissemination and tumour location. *International Journal of Oncology.*, **15,** 491-8.

Gonen, M., Hummer, A., Zervoudakis, A., Sullivan, D., Fong, Y., Banerjee, D., Klimstra, D., Cordon-Cardo, C., Bertino, J. & Kemeny, N. (2003). Thymidylate synthase expression in hepatic tumors is a predictor of survival and progression in patients with resectable metastatic colorectal cancer.[see comment]. *Journal of Clinical Oncology*, **21,** 406-12.

Gonzalez-Aguilera, J.J., Oliart, S., Azcoita, M.M. & Fernandez-Peralta, A.M. (2004). Simultaneous mutations in K-ras and TP53 are indicative of poor prognosis in sporadic colorectal cancer. *American Journal of Clinical Oncology*, **27,** 39-45.

Grewal, H., Guillem, J.G., Klimstra, D.S. & Cohen, A.M. (1995). p53 nuclear overexpression may not be an independent prognostic marker in early colorectal cancer. *Diseases of the Colon and Rectum*, **38,** 1176-1181.

Gunther, K., Dworak, O., Remke, S., Pfluger, R., Merkel, S., Hohenberger, W. & Reymond, M.A. (2002). Prediction of distant metastases after curative surgery for rectal cancer. *Journal of Surgical Research.*, **103,** 68-78.

Hallak, R., Mueller, J., Lotter, O., Gansauge, S., Gansauge, F., Jumma, M.-D., Montenarh, M., Safi, F. & Beger, H. (1998). p53 genetic alterations, protein expression and autoantibodies in human colorectal carcinoma: A comparative study. *International Journal of Oncology.*, **12,** 785-791.

Hamelin, R., Laurent-Puig, P., Olschwang, S., Jego, N., Asselain, B., Remvikos, Y., Girodet, J., Salmon, R.J. & Thomas, G. (1994). Association of p53 mutations with short survival in colorectal cancer. *Gastroenterology.*, **106,** 42-48.

Hardingham, J.E., Butler, W.J., Roder, D., Dobrovic, A., Dymock, R.B., Sage, R.E. & Roberts-Thomson, I.C. (1998). Somatic mutations, acetylator status, and prognosis in colorectal cancer. *Gut.*, **42,** 669-72.

Haseba, M., Hidaka, S., Tsuji, T., Yano, H., Komatsu, H., Sawai, T., Yasutake, T., Nakagoe, T., Tagawa, Y. & Ayabe, H. (2003). Detection of p53 Gene Mutations by Nonisotopic RNase Cleavage Assay as a Predictor of Poor Prognosis in Colorectal Cancers. *Digestive Diseases & Sciences*, **48,** 1984-1989.

Hayashi, N., Ito, I., Yanagisawa, A., Kato, Y., Nakamori, S., Imaoka, S., Watanabe, H., Ogawa, M. & Nakamura, Y. (1995). Genetic diagnosis of lymph-node metastasis in colorectal cancer.[comment]. *Lancet.*, **345,** 1257-9.

Heisterkamp, J., van Bommel, J., Hop, W.C., Tilanus, H.W., Zondervan, P.E. & JN, I.J. (1999). P53 overexpression in colorectal metastases confined to the liver and outcome of liver resection. *Hepato-Gastroenterology.*, **46,** 3109-14.

Hirano, K. & Minamoto, T. (2000). Altered expression of p53 and p27 proteins, alone or combined, as a predictor of metastatic potential in early invasive carcinoma of colon and rectum--a comparative clinicopathologic and molecular analysis. *Cancer Detection & Prevention.*, **24,** 343-55.

Hirvikoski, P., Auvinen, A., Servomaa, K., Kiuru, A., Rytomaa, T., Makkonen, K. & Kosma, V.M. (1999). K-ras and p53 mutations and overexpressions as prognostic factors in female rectal carcinoma. *Anticancer Research.*, **19,** 685-691.

Ichikawa, W. (1994). Positive relationship between expression of CD44 and hepatic metastases in colorectal cancer. *Pathobiology.*, **62,** 172-9.

Ilyas, M., Hao, X.P., Wilkinson, K., Tomlinson, I.P., Abbasi, A.M., Forbes, A., Bodmer, W.F. & Talbot, I.C. (1998). Loss of Bcl-2 expression correlates with tumour recurrence in colorectal cancer. *Gut.*, **43,** 383-7.

Iniesta, P., Vega, F.J., Caldes, T., Massa, M., de Juan, C., Cerdan, F.J., Sanchez, A., Lopez, J.A., Torres, A.J., Balibrea, J.L. & Benito, M. (1998). p53 exon 7 mutations as a predictor of poor prognosis in patients with colorectal cancer. *Cancer Letters.*, **130,** 153-60.

Jansson, A., Gentile, M. & Sun, X.F. (2001). P53 mutations are present in colorectal cancer with cytoplasmic p53 accumulation. *International Journal of Cancer.*, **92,** 338-341.

Jingwei, Z., Yixian, L. & Zhijiao, T. (1998). Study of the relation between P53 expression and pathological factors in colorectal carcinoma and it's prognostic significance. [Chinese]. *Acta Academiae Medicinae Hubei.*, **19,** 7-9+15.

Kahlenberg, M.S., Stoler, D.L., Rodriguez-Bigas, M.A., Weber, T.K., Driscoll, D.L., Anderson, G.R. & Petrelli, N.J. (2000). p53 tumor suppressor gene mutations predict decreased survival of patients with sporadic colorectal carcinoma. *Cancer.*, **88,** 1814-1819.

Kandioler, D., Zwrtek, R., Ludwig, C., Janschek, E., Ploner, M., Hofbauer, F., Kuhrer, I., Kappel, S., Wrba, F., Horvath, M., Karner, J., Renner, K., Bergmann, M., Karner_Hanusch, J., Potter, R., Jakesz, R., Teleky, B. & Herbst, F. (2002). TP53 genotype but not p53 immunohistochemical result predicts response to preoperative short-term radiotherapy in rectal cancer. *Annals of Surgery*, **235,** 493-8.

Kang, S.M., Maeda, K., Onoda, N., Chung, Y.S., Nakata, B., Nishiguchi, Y. & Sowa, M. (1997). Combined analysis of p53 and vascular endothelial growth factor expression in colorectal carcinoma for determination of tumor vascularity and liver metastasis. *International Journal of Cancer.*, **74,** 502-507.

Kapiteijn, E., Liefers, G.J., Los, L.C., Kranenbarg, E.K., Hermans, J., Tollenaar, R., Moriya, Y., Van de Velde, C.J.H. & Van Krieken, J. (2001). Mechanisms of oncogenesis in colon versus rectal cancer. *Journal of Pathology.*, **195,** 171-178.

Khan, Z.A., Jonas, S.K., Feldmann, K.A., Patel, H., Wharton, R.Q., Tarragona, A., Ivison, A. & Allen-Mersh, T.G. (2001). P53 mutation and response to hepatic arterial floxuridine in patients with colorectal liver metastases. *Journal of Cancer Research & Clinical Oncology.*, **127,** 675-80.

Kim, Y.W., Lee, S., Park, J.H., Yoon, T.Y., Park, Y.K. & Yang, M.H. (1995). An immunohistochemical study of the expression of p53 protein in colon cancer. *Journal of Korean Medical Science.*, **10,** 176-82.

Klump, B., Nehls, O., Okech, T., Hsieh, C.J., Gaco, V., Gittinger, F.S., Sarbia, M., Borchard, F., Greschniok, A., Gruenagel, H.H., Porschen, R. & Gregor, M. (2004). Molecular lesions in colorectal cancer: Impact on prognosis? Original data and review of the literature. *International Journal of Colorectal Disease*, **19,** 23-42.

Kotera, H., Kusunoki, M., Yanagi, H., Yamamura, T. & Utsunomiya, J. (1997). A possible correlation between p53 overexpression and prognosis after radiotherapy combined surgery for rectal carcinoma. *International Journal of Oncology.*, **10,** 971-975.

Kressner, U., Inganas, M., Byding, S., Blikstad, I., Pahlman, L., Glimelius, B. & Lindmark, G. (1999). Prognostic value of p53 genetic changes in colorectal cancer. *Journal of Clinical Oncology.*, **17,** 593-9.

Kressner, U., Lindmark, G., Gerdin, B., Pahlman, L. & Glimelius, B. (1996). Immunohistochemical p53 staining is of limited value in the staging and prognostic prediction of colorectal cancer. *Anticancer Research.*, **16,** 951-957.

Lanza, G., Jr., Maestri, I., Dubini, A., Gafa, R., Santini, A., Ferretti, S. & Cavazzini, L. (1996). p53 expression in colorectal cancer: relation to tumor type, DNA ploidy pattern and short-term survival. *American Journal of Clinical Pathology.*, **105,** 604-12.

Lashner, B.A., Bauer, W.M., Rybicki, L.A. & Goldblum, J.R. (2003). Abnormal p53 immunohistochemistry is associated with an increased colorectal cancer-related mortality in patients with ulcerative colitis. *American Journal of Gastroenterology*, **98,** 1423-1427.

Lazaris, A.C., Theodoropoulos, G.E., Anastassopoulos, P., Nakopoulou, L., Panoussopoulos, D. & Papadimitriou, K. (1995). Prognostic significance of p53 and c-erbB-2 immunohistochemical evaluation in colorectal adenocarcinoma. *Histology & Histopathology.*, **10,** 661-668.

Leahy, D.T., Salman, R., Mulcahy, H., Sheahan, K., O'Donoghue, D.P. & Parfrey, N.A. (1996). Prognostic significance of p53 abnormalities in colorectal carcinoma detected by PCR-SSCP and immunohistochemical analysis. *Journal of Pathology.*, **180,** 364-370.

Lenz, H.J., Hayashi, K., Salonga, D., Danenberg, K.D., Danenberg, P.V., Metzger, R., Banerjee, D., Bertino, J.R., Groshen, S., Leichman, L.P. & Leichman, C.G. (1998). p53 point mutations and thymidylate synthase messenger RNA levels in disseminated colorectal cancer: an analysis of response and survival. *Clinical Cancer Research : An Official Journal of the American Association For Cancer Research*, **4,** 1243-50.

Liang, J.T., Chang, K.J., Chen, J.C., Lee, C.C., Cheng, Y.M., Hsu, H.C., Chien, C.T. & Wang, S.M. (1999a). Clinicopathologic and carcinogenetic appraisal of DNA replication error in sporadic T3N0M0 stage colorectal cancer after curative resection. *Hepato-Gastroenterology.*, **46,** 883-890.

Liang, J.T., Cheng, Y.M., Chang, K.J., Chien, C.T. & Hsu, H.C. (1999b). Reappraisal of K-ras and p53 gene mutations in the recurrence of Dukes' B2 rectal cancer after curative resection. *Hepato-Gastroenterology*, **46,** 830-7.

Liang, J.T., Huang, K.C., Cheng, A.L., Jeng, Y.M., Wu, M.S. & Wang, S.M. (2003). Clinicopathological and molecular biological features of colorectal cancer in patients less than 40 years of age. *The British Journal of Surgery*, **90,** 205-14.

Liang, J.T., Huang, K.C., Cheng, Y.M., Hsu, H.C., Cheng, A.L., Hsu, C.H., Yeh, K.H., Wang, S.M. & Chang, K.J. (2002a). P53 overexpression predicts poor chemosensitivity to high-dose 5-fluorouracil plus leucovorin chemotherapy for stage IV colorectal cancers after palliative bowel resection. *International Journal of Cancer. Journal International Du Cancer*, **97,** 451-7.

Liang, J.T., Huang, K.C., Jeng, Y.M., Lee, P.H., Lai, H.S. & Hsu, H.C. (2004). Microvessel density, cyclo-oxygenase 2 expression, K-ras mutation and p53 overexpression in colonic cancer. *The British Journal of Surgery*, **91,** 355-61.

Liang, J.T., Huang, K.C., Lai, H.S., Lee, P.H., Cheng, Y.M., Hsu, H.C., Cheng, A.L., Hsu, C.H., Yeh, K.H., Wang, S.M., Tang, C. & Chang, K.J. (2002b). High-frequency microsatellite instability predicts better chemosensitivity to high-dose 5-fluorouracil plus leucovorin chemotherapy for stage IV sporadic colorectal cancer after palliative bowel resection. *International Journal of Cancer. Journal International Du Cancer*, **101,** 519-25.

Lopez-Guerrero, J.A., Gilabert, P.B. & Gonzalez, E.B. (1997). Biological and oncological significance of the molecular alterations of p53 in human cancer. [Spanish]. *Oncologia*, **20,** 25-38.

Luna-Perez, P., Arriola, E.L., Cuadra, Y., Alvarado, I. & Quintero, A. (1998). p53 protein overexpression and response to induction chemoradiation therapy in patients with locally advanced rectal adenocarcinoma. *Annals of Surgical Oncology.*, **5,** 203-8.

Maeda, K., Chung, Y.S., Kang, S.M., Ogawa, M., Onoda, N., Nakata, B., Nishiguchi, Y., Ikehara, T., Okuno, M. & Sowa, M. (1997). Overexpression of cyclin D1 and p53 associated with disease recurrence in colorectal adenocarcinoma. *International Journal of Cancer.*, **74,** 310-315.

Manne, U., Myers, R.B., Moron, C., Poczatek, R.B., Dillard, S., Weiss, H., Brown, D., Srivastava, S. & Grizzle, W.E. (1997). Prognostic significance of Bcl-2 expression and p53 nuclear accumulation in colorectal adenocarcinoma. *International Journal of Cancer.*, **74,** 346-358.

Manne, U., Weiss, H.L., Myers, R.B., Danner, O.K., Moron, C., Srivastava, S. & Grizzle, W.E. (1998). Nuclear accumulation of p53 in colorectal adenocarcinoma prognostic importance differs with race and location of the tumor. *Cancer.*, **83,** 2456-2467.

Mulder, J.W., Baas, I.O., Polak, M.M., Goodman, S.N. & Offerhaus, G.J. (1995). Evaluation of p53 protein expression as a marker for long-term prognosis in colorectal carcinoma. *British Journal of Cancer.*, **71,** 1257-62.

Nasierowska-Guttmejer, A. (2001). The comparison of immunohistochemical proliferation and apoptosis markers in rectal carcinoma treated surgically or by preoperative radio-chemotherapy. *Polish Journal of Pathology.*, **52,** 53-61.

Nathanson, S.D., Linden, M.D., Tender, P., Zarbo, R.J., Jacobsen, G. & Nelson, L.T. (1994). Relationship among p53, stage, and prognosis of large bowel cancer. *Diseases of the Colon and Rectum*, **37,** 527-534.

Nehls, O., Klump, B., Holzmann, K., Lammering, G., Borchard, F., Gruenagel, H.H., Gaco, V., Gregor, M. & Porschen, R. (1999). Influence of p53 status on prognosis in preoperatively irradiated rectal carcinoma. *Cancer.*, **85,** 2541-2548.

Nitti, D., Belluco, C., Montesco, M.C., Bertorelle, R., Da_Pian, P.P., Fassina, A., Ninfo, V., Chieco_Bianchi, L. & Lise, M. (1998). Nuclear p53 protein expression in resected hepatic metastases from colorectal cancer: an independent prognostic factor of survival. *European Journal of Cancer (Oxford, England : 1990)*, **34,** 851-5.

Ofner, D., Maier, H., Riedmann, B., Holzberger, P., Nogler, M., Totsch, M., Banfalvi, A., Winde, G., Bocker, W. & Schmid, K.W. (1995). Immunohistochemically detectable p53 and mdm-2 oncoprotein expression in colorectal carcinoma: Prognostic significance. *Journal of Clinical Pathology: Clinical Molecular Pathology.*, **48,** M12-M16.

Ofner, D. & Schmid, K.P. (1994). Prognostic relevance of p53 mutation in colorectal carcinoma.[comment]. *Gastroenterology.*, **107,** 1571-2.

Ogiwara, H., Nakamura, T. & Baba, S. (1994). Variables related to risk of recurrence in rectal cancer without lymph node metastasis.[comment]. *Annals of Surgical Oncology.*, **1,** 99-104.

Okonkwo, A., Musunuri, S., Talamonti, M., Benson, A., Small, W., Stryker, S.J. & Rao, M.S. (2001). Molecular markers and prediction of response to chemoradiation in rectal cancer. *Oncology Reports*, **8,** 497-500.

Okubo, R., Masuda, H. & Nemoto, N. (2001). p53 mutation found to be a significant prognostic indicator in distal colorectal cancer. *Oncology Reports.*, **8,** 509-14.

Paradiso, A., Ranieri, G., Simone, G., Silvestris, N., Costa, A., De Lena, M., Leone, A., Vallejo, C. & Lacava, J. (2002). mdm2-p53 interaction: Lack of correlation with the response to 5-fluorouracil in advanced colorectal cancer. *Oncology.*, **62,** 278-285.

Paradiso, A., Simone, G., Petroni, S., Leone, B., Vallejo, C., Lacava, J., Romero, A., Machiavelli, M., De Lena, M., Allegra, C.J. & Johnston, P.G. (2000). Thymidilate synthase and p53 primary tumour expression as predictive factors for advanced colorectal cancer patients. *British Journal of Cancer.*, **82,** 560-567.

Pavillard, V., Charasson, V., Laroche-Clary, A., Soubeyran, I. & Robert, J. (2004). Cellular parameters predictive of the clinical response of colorectal cancers to irinotecan. A preliminary study. *Anticancer Research*, **24,** 579-85.

Pereira, H., Silva, S., Juliao, R., Garcia, P. & Perpetua, F. (1997). Prognostic markers for colorectal cancer: Expression of P53 and BCL2. *World Journal of Surgery.*, **21,** 210-213.

Pocard, M., Paradis, V., Adam, R., Reynes, M. & Bismuth, H. (1998). Possible prognostic significance of p53 immunoreactive status of hepatic colorectal cancer metastases following surgical resection. *European Journal of Cancer.*, **34,** 594-5.

Poller, D.N., Baxter, K.J. & Shepherd, N.A. (1997). p53 and Rb1 protein expression: Are they prognostically useful in colorectal cancer? *British Journal of Cancer.*, **75,** 87-93.

Pricolo, V.E., Finkelstein, S.D., Hansen, K., Cole, B.F., Bland, K.I., Jessup, J., Kern, K.A. & Rattner, D.W. (1997). Mutated p53 gene is an independent adverse predictor of survival in colon carcinoma. *Archives of Surgery.*, **132,** 371-375.

Pricolo, V.E., Finkelstein, S.D., Wu, T.T., Keller, G., Bakker, A., Swalsky, P.A. & Bland, K.I. (1996). Prognostic value of TP53 and K-ras-2 mutational analysis in stage III carcinoma of the colon. *American Journal of Surgery.*, **171,** 41-6.

Qiu, H., Sirivongs, P., Rothenberger, M., Rothenberger, D.A. & Garcia-Aguilar, J. (2000). Molecular prognostic factors in rectal cancer treated by radiation and surgery. *Diseases of the Colon and Rectum*, **43,** 451-459.

Rau, B., Sturm, I., Lage, H., Berger, S., Schneider, U., Hauptmann, S., Wust, P., Riess, H., Schlag, P.M., Dorken, B. & Daniel, P.T. (2003). Dynamic expression profile of p21WAF1/CIP1 and Ki-67 predicts survival in rectal carcinoma treated with preoperative radiochemotherapy.[see comment]. *Journal of Clinical Oncology*, **21,** 3391-401.

Rebischung, C., Gerard, J.P., Gayet, J., Thomas, G., Hamelin, R. & Laurent-Puig, P. (2002). Prognostic value of P53 mutations in rectal carcinoma. *International Journal of Cancer.*, **100,** 131-135.

Remvikos, Y., Tominaga, O., Hammel, P., Laurent-Puig, P., Salmon, R.J., Dutrillaux, B. & Thomas, G. (1992). Increased p53 protein content of colorectal tumours correlates with poor survival. *British Journal of Cancer.*, **66,** 758-764.

Rew, D.A., Karkera, R., Mullee, M.A., Julious, S.A. & Wilson, G.D. (1996). The flow cytometric analysis of total p53 protein content and proliferation indices in colorectal cancer, in relation to clinical outcome. *European Journal of Surgical Oncology.*, **22,** 508-515.

Rodel, C., Grabenbauer, G.G., Papadopoulos, T., Bigalke, M., Gunther, K., Schick, C., Peters, A., Sauer, R. & Rodel, F. (2002). Apoptosis as a cellular predictor for histopathologic response to neoadjuvant radiochemotherapy in patients with rectal cancer. *International Journal of Radiation Oncology, Biology, Physics.*, **52,** 294-303.

Rosty, C., Chazal, M., Etienne, M.C., Letoublon, C., Bourgeon, A., Delpero, J.R., Pezet, D., Beaune, P., Laurent-Puig, P. & Milano, G. (2001). Determination of microsatellite instability, p53 and K-ras mutations in hepatic metastases from patients with colorectal cancer: Relationship with response to 5-fluorouracil and survival. *International Journal of Cancer.*, **95,** 162-167.

Russo, A., Migliavacca, M., Bazan, V., Maturi, N., Morello, V., Dardanoni, G., Modica, G., Bazan, P., Albanese, I., La Farina, M. & Tomasino, R.M. (1998). Prognostic significance of proliferative activity, DNA-ploidy, p53 and Ki-ras point mutations in colorectal liver metastases. *Cell Proliferation.*, **31,** 139-153.

Russo, A., Migliavacca, M., Zanna, I., Valerio, M.R., Latteri, M.A., Grassi, N., Pantuso, G., Salerno, S., Dardanoni, G., Albanese, I., La Farina, M., Tomasino, R.M., Gebbia, N. & Bazan, V. (2002). p53 mutations in L3-loop zinc-binding domain, DNA-ploidy, and S phase fraction are independent

prognostic indicators in colorectal cancer: A prospective study with a five-year follow-up. *Cancer Epidemiology, Biomarkers & Prevention.*, **11,** 1322-1331.

Sakakura, C., Koide, K., Ichikawa, D., Wakasa, T., Shirasu, M., Kimura, A., Taniguchi, H., Hagiwara, A., Yamaguchi, T., Inazawa, J., Abe, T., Takahashi, T. & Otsuji, E. (1998). Analysis of histological therapeutic effect, apoptosis rate and p53 status after combined treatment with radiation, hyperthermia and 5-fluorouracil suppositories for advanced rectal cancers. *British Journal of Cancer.*, **77,** 159-66.

Samowitz, W.S., Curtin, K., Ma, K.N., Edwards, S., Schaffer, D., Leppert, M.F. & Slattery, M.L. (2002). Prognostic significance of p53 mutations in colon cancer at the population level. *International Journal of Cancer.*, **99,** 597-602.

Saw, R.P., Koorey, D., Painter, D., Gallagher, P.J. & Solomon, M.J. (2002). p53, DCC and thymidylate synthase as predictors of survival after resection of hepatic metastases from colorectal cancer. *British Journal of Surgery.*, **89,** 1409-15.

Saw, R.P.M., Morgan, M., Koorey, D., Painter, D., Findlay, M., Stevens, G., Clarke, S., Chapuis, P. & Solomon, M.J. (2003). p53, deleted in colorectal cancer gene, and thymidylate synthase as predictors of histopathologic response and survival in low, locally advanced rectal cancer treated with preoperative adjuvant therapy. *Diseases of the Colon & Rectum*, **46,** 192-202.

Schelwies, K., Sturm, I., Grabowski, P., Scherubl, H., Schindler, I., Hermann, S., Stein, H., Buhr, H.J., Riecken, E.O., Zeitz, M., Dorken, B. & Daniel, P.T. (2002). Analysis of p53/BAX in primary colorectal carcinoma: Low BAX protein expression is a negative prognostic factor in UICC stage III tumors. *International Journal of Cancer.*, **99,** 589-596.

Schwandner, O., Bruch, H.P. & Broll, R. (2002). p21, p27, cyclin D1, and p53 in rectal cancer: immunohistology with prognostic significance? *International Journal of Colorectal Disease.*, **17,** 11-9.

Schwandner, O., Schiedeck, T.H., Bruch, H.P., Duchrow, M., Windhoevel, U. & Broll, R. (2000). p53 and Bcl-2 as significant predictors of recurrence and survival in rectal cancer. *European Journal of Cancer.*, **36,** 348-56.

Scott, N., Sagar, P., Stewart, J., Blair, G.E., Dixon, M.F. & Quirke, P. (1991). p53 in colorectal cancer: Clinicopathological correlation and prognostic significance. *British Journal of Cancer.*, **63,** 317-319.

Silverstrini, R. & Costa, A. (1995). Cell proliferation and related genes as indicators of prognosis in colorectal cancers. *Journal of Experimental & Clinical Cancer Research.*, **14,** 9-13.

Silvestrini, R., Costa, A. & Faranda, A. (1996). Biological prospectives to define prognosis and treatment strategies in liver metastases from colorectal cancer. *Annali Italiani di Chirurgia.*, **67,** 733-7.

Slebos, R.J.C., Baas, I.O., Clement, M., Polak, M., Mulder, J.W., Van den Berg, F.M., Hamilton, S.R. & Offerhaus, G.J.A. (1996). Clinical and pathological associations with p53 tumour-suppressor gene mutations and expression of p21(WAF1/Cip1) in colorectal carcinoma. *British Journal of Cancer*, **74,** 165-171.

Smith, D.R. & Goh, H.S. (1996). p53 and prognosis in colorectal cancer. *Annals of the Academy of Medicine, Singapore.*, **25,** 107-12.

Soong, R., Grieu, F., Robbins, P., Dix, B., Chen, D., Parsons, R., House, A. & Iacopetta, B. (1997). p53 Alterations are associated with improved prognosis in distal colonic carcinomas. *Clinical Cancer Research.*, **3,** 1405-1411.

Soong, R., Powell, B., Elsaleh, H., Gnanasampanthan, G., Smith, D.R., Goh, H.S., Joseph, D. & Iacopetta, B. (2000). Prognostic significance of TP53 gene mutation in 995 cases of colorectal carcinoma. Influence of tumour site, stage, adjuvant chemotherapy and type of mutation. *European Journal of Cancer.*, **36,** 2053-60.

Sory, A., Minamoto, T., Ohta, T., Yamashita, K., Sawaguchi, K., Mai, M. & Misiuna, P. (1997). Does p53 overexpression cause metastases in early invasive colorectal adenocarcinoma? *European Journal of Surgery.*, **163,** 685-692.

Spitz, F.R., Giacco, G.G., Hess, K., Larry, L., Rich, T.A., Janjan, N., Cleary, K.R. & Skibber, J.M. (1997). p53 immunohistochemical staining predicts residual disease after chemoradiation in patients with high-risk rectal cancer. *Clinical Cancer Research : An Official Journal of the American Association For Cancer Research*, **3,** 1685-90.

Starzynska, T., Bromley, M., Ghosh, A. & Stern, P.L. (1992). Prognostic significance of p53 overexpression in gastric and colorectal carcinoma. *British Journal of Cancer.*, **66,** 558-562.

Starzynska, T., Bromley, M., Marlicz, K., Roberts, S.A., Ucinski, M. & Stern, P.L. (1997). Accumulation of p53 in relation to long-term prognosis in colorectal carcinoma. *European Journal of Gastroenterology & Hepatology.*, **9,** 183-186.

Sturm, I., Kohne, C.H., Wolff, G., Petrowsky, H., Hillebrand, T., Hauptmann, S., Lorenz, M., Dorken, B. & Daniel, P.T. (1999). Analysis of the p53/BAX pathway in colorectal cancer: Low BAX is a negative prognostic factor in patients with resected liver metastases. *Journal of Clinical Oncology.*, **17,** 1364-1374.

Subramaniam, P., Sinha, S.N. & Wilkinson, S. (1995). Immunohistochemical detection of p53 and the recurrence of rectal adenocarcinoma. *The Australian and New Zealand Journal of Surgery*, **65,** 740-2.

Sun, X.F., Carstensen, J.M. & Nordenskjold, B. (1995). Expression of c-erbB-2 and p53 in colorectal adenocarcinoma. *Analytical Cellular Pathology.*, **8,** 203-211.

Sun, X.F., Carstensen, J.M., Stal, O., Zhang, H., Nilsson, E., Sjodahl, R. & Nordenskjold, B. (1993). Prognostic significance of p53 expression in relation to DNA ploidy in colorectal adenocarcinoma. *Virchows Archiv - A, Pathological Anatomy & Histopathology.*, **423,** 443-448.

Sun, X.F., Carstensen, J.M., Zhang, H., Arbman, G. & Nordenskjold, B. (1996). Prognostic significance of p53 nuclear and cytoplasmic overexpression in right and left colorectal adenocarcinomas. *European Journal of Cancer Part A*, **32,** 1963-1967.

Sun, X.F., Zhang, H., Carstensen, J., Jansson, A. & Nordenskjold, B. (1997). Heat shock protein 72/73 in relation to cytoplasmic p53 expression and prognosis in colorectal adenocarcinomas. *International Journal of Cancer.*, **74,** 600-604.

Suzuki, H., Matsumoto, K., Koide, A., Tada, T., Fujino, I., Okuda, A. & Shigemori, C. (1994). Correlation of p53 with the clinicopathologic features and prognosis of colorectal adenocarcinoma. *Surgery Today.*, **24,** 85-87.

Tollenaar, R.A.E., Van Krieken, J.H.J., Van Slooten, H.J., Bruinvels, D.J., Nelemans, K.M.J., Van den Broek, L.J., Hermans, J. & Van Dierendonck, J.H. (1998). Immunohistochemical detection of p53 and Bcl-2 in colorectal carcinoma: No evidence for prognostic significance. *British Journal of Cancer.*, **77,** 1842-1847.

Tortola, S., Marcuello, E., Gonzalez, I., Reyes, G., Arribas, R., Aiza, G., Sancho, F.J., Peinado, M.A. & Capella, G. (1999). p53 and K-ras gene mutations correlate with tumor aggressiveness but are not of routine prognostic value in colorectal cancer. *Journal of Clinical Oncology.*, **17,** 1375-1381.

Tullo, A., D'Erchia, A.M., Honda, K., Mitry, R.R., Kelly, M.D., Habib, N.A., Saccone, C. & Sbisa, E. (1999). Characterization of p53 mutations in colorectal liver metastases and correlation with clinical parameters. *Clinical Cancer Research.*, **5,** 3523-3528.

Visca, P., Alo, P.L., Del Nonno, F., Botti, C., Trombetta, G., Marandino, F., Filippi, S., Di Tondo, U. & Donnorso, R.P. (1999). Immunohistochemical expression of fatty acid synthase, apoptotic- regulating genes, proliferating factors, and ras protein product in colorectal adenomas, carcinomas, and adjacent nonneoplastic mucosa. *Clinical Cancer Research*, **5,** 4111-4118.

Wang, Q., Zhao, P., Hu, Y. & Yang, G. (1998). Prognostic significance of P53 protein expression in patients with colorectal adenocarcinoma. *Hua-Hsi i Ko Ta Hsueh Hsueh Pao [Journal of West China University of Medical Sciences]*. **29,** 182-4.

Watanabe, T., Wu, T.T., Catalano, P.J., Ueki, T., Satriano, R., Haller, D.G., Benson, A.B. & Hamilton, S.R. (2001). Molecular predictors of survival after adjuvant chemotherapy for colon cancer. *The New England Journal of Medicine*, **344,** 1196-206.

Wiggenraad, R., Tamminga, R., Blok, P., Rouse, R. & Hermans, J. (1998). The prognostic significance of p53 expression for survival and local control in rectal carcinoma treated with surgery and postoperative radiotherapy. *International Journal of Radiation Oncology, Biology, Physics.*, **41,** 29-35.

Yamaguchi, A., Kurosaka, Y., Fushida, S., Kanno, M., Yonemura, Y., Miwa, K. & Miyazaki, I. (1992). Expression of p53 protein in colorectal cancer and its relationship to short-term prognosis. *Cancer.*, **70,** 2778-2784.

Yamaguchi, A., Nakagawara, G., Kurosaka, Y., Nishimura, G., Yonemura, Y. & Miyazaki, I. (1993). P53 immunoreaction in endoscopic biopsy specimens of colorectal cancer, and its prognostic significance. *British Journal of Cancer.*, **68,** 399-402.

Yamamura, T., Matsuzaki, H., Suda, T., Ozasa, T., Tsukikawa, S. & Yamaguchi, S. (1999). Clinicopathological variables and p53 overexpression as a combined prognosticator for hematogenic recurrence in colorectal cancer. *Journal of Surgical Oncology.*, **70,** 1-5.

Yang, Y., Forslund, A., Remotti, H., Lonnroth, C., Andersson, M., Brevinge, H., Svanberg, E., Lindner, P., Hafstrom, L., Naredi, P. & Lundholm, K. (2001). P53 mutations in primary tumors and subsequent liver metastases are related to survival in patients with colorectal carcinoma who undergo liver resection. *Cancer.*, **91,** 727-36.

Yoshikawa, R., Yanagi, H., Kusunoki, M., Fujiwara, Y., Noda, M., Hashimoto_Tamaoki, T. & Yamamura, T. (2002). Prognostic values of radiation-induced p53 in adjacent normal mucosa and p21WAF1/CIP1 expression in rectal cancer patients. *International Journal of Oncology*, **21,** 1223-8.

Zeng, Z.S., Sarkis, A.S., Zhang, Z.F., Klimstra, D.S., Charytonowicz, E., Guillem, J.G., Cordon-Cardo, C. & Cohen, A.M. (1994). p53 nuclear overexpression: an independent predictor of survival in lymph node--positive colorectal cancer patients. *Journal of Clinical Oncology.*, **12,** 2043-50.

Zhang, H. (1999). Evaluation of four antibodies in detecting p53 protein for predicting clinicopathological and prognostic significance in colorectal adenocarcinoma. *Clinical Cancer Research.*, **5,** 4126-4132.

Zirbes, T.K., Baldus, S.E., Moenig, S.P., Nolden, S., Kunze, D., Shafizadeh, S.T., Schneider, P.M., Thiele, J., Hoelscher, A.H. & Dienes, H.P. (2000). Prognostic impact of p21/waf1/cip1 in colorectal cancer. *International Journal of Cancer.*, **89,** 14-18.
